# Single-domain magnetic particles with motion behavior under electromagnetic AC and DC fields are a fatal cargo in Metropolitan Mexico City pediatric and young adult early Alzheimer, Parkinson, frontotemporal lobar degeneration and amyotrophic lateral sclerosis and in ALS patients

**DOI:** 10.3389/fnhum.2024.1411849

**Published:** 2024-08-23

**Authors:** Lilian Calderón-Garcidueñas, Fredy Rubén Cejudo-Ruiz, Elijah W. Stommel, Angélica González-Maciel, Rafael Reynoso-Robles, Ricardo Torres-Jardón, Samuel Tehuacanero-Cuapa, Arturo Rodríguez-Gómez, Francisco Bautista, Avto Goguitchaichvili, Beatriz E. Pérez-Guille, Rosa Eugenia Soriano-Rosales, Emel Koseoglu, Partha S. Mukherjee

**Affiliations:** ^1^Department of Biomedical Sciences, The University of Montana, Missoula, MT, United States; ^2^Instituto de Geofísica, Universidad Nacional Autónoma de México, Mexico City, Mexico; ^3^Department of Neurology, Geisel School of Medicine at Dartmouth, Hanover, NH, United States; ^4^Instituto Nacional de Pediatría, Mexico City, Mexico; ^5^Instituto de Ciencias de la Atmósfera y Cambio Climático, Universidad Nacional Autónoma de México, Mexico City, Mexico; ^6^Instituto de Física, Universidad Nacional Autónoma de México, Mexico City, Mexico; ^7^Centro de Investigaciones en Geografía Ambiental, Universidad Nacional Autónoma de México, Morelia, Michoacan, Mexico; ^8^Department of Neurology, Erciyes Faculty of Medicine, Erciyes University, Kayseri, Türkiye; ^9^Interdisciplinary Statistical Research Unit, Indian Statistical Institute, Kolkata, India

**Keywords:** brain magnetic nanoparticles, saturation isothermal remanent magnetization SIRM, pediatric Alzheimer, single domain FeNPs, Motion nanoparticle behavior, Alzheimer, Parkinson, frontotemporal lobar degeneration

## Abstract

Metropolitan Mexico City (MMC) children and young adults exhibit overlapping Alzheimer and Parkinsons’ diseases (AD, PD) and TAR DNA-binding protein 43 pathology with magnetic ultrafine particulate matter (UFPM) and industrial nanoparticles (NPs). We studied magnetophoresis, electron microscopy and energy-dispersive X-ray spectrometry in 203 brain samples from 14 children, 27 adults, and 27 ALS cases/controls. Saturation isothermal remanent magnetization (SIRM), capturing magnetically unstable FeNPs ~ 20nm, was higher in caudate, thalamus, hippocampus, putamen, and motor regions with subcortical vs. cortical higher SIRM in MMC ≤ 40y. Motion behavior was associated with magnetic exposures 25–100 mT and children exhibited IRM saturated curves at 50–300 mT associated to change in NPs position and/or orientation *in situ*. Targeted magnetic profiles moving under AC/AD magnetic fields could distinguish ALS vs. controls. Motor neuron magnetic NPs accumulation potentially interferes with action potentials, ion channels, nuclear pores and enhances the membrane insertion process when coated with lipopolysaccharides. TEM and EDX showed 7–20 nm NP Fe, Ti, Co, Ni, V, Hg, W, Al, Zn, Ag, Si, S, Br, Ce, La, and Pr in abnormal neural and vascular organelles. Brain accumulation of magnetic unstable particles start in childhood and cytotoxic, hyperthermia, free radical formation, and NPs motion associated to 30–50 μT (DC magnetic fields) are critical given ubiquitous electric and magnetic fields exposures could induce motion behavior and neural damage. Magnetic UFPM/NPs are a fatal brain cargo in children’s brains, and a preventable AD, PD, FTLD, ALS environmental threat. Billions of people are at risk. We are clearly poisoning ourselves.

## 1 Introduction

Magnetic nanoscale particulates, including ultrafine particulate matter (UFPM) from anthropogenic combustion-friction sources and industrial nanoparticles (NPs) are present in the brains of children, young adults, fetuses and their placentas in highly polluted urban environments (Calderón-Garcidueñas et al., 2022a; Calderón-Garcidueñas et al., 2017; Calderón-Garcidueñas and Ayala, 2022; Calderón-Garcidueñas et al., 2022b; Calderón-Garcidueñas et al., 2021; Calderón-Garcidueñas et al., 2018a; Calderón-Garcidueñas et al., 2018b; Calderón-Garcidueñas et al., 2020; Calderón-Garcidueñas et al., 2024a; Calderón-Garcidueñas et al., 2024b. Toxic anthropogenic metal, metalloid and natural nano size particles are associated with significant subcellular, neural and cardiac atrioventricular conduction axis ultrastructural pathology and breaking of the neurovascular unit (Calderón-Garcidueñas et al., 2022a; Calderón-Garcidueñas et al., 2017; Calderón-Garcidueñas and Ayala, 2022; Calderón-Garcidueñas et al., 2022b; Calderón-Garcidueñas et al., 2021). Metropolitan Mexico City (MMC) 22 million residents have been exposed for decades to high concentrations of fine particulate matter PM_2.5_, UFPM, and NPs (Aguilar-Castillo, 2023; Velasco et al., 2019; Caudillo et al., 2020; Dunn et al., 2004; Kleinman et al., 2009). We published in 2004, the first work establishing the association between long-term exposure to PM_2.5_ and neuroinflammation and Alzheimer pathology in young MMC urbanites (Calderón-Garcidueñas et al., 2004). The development and progression of the neuropathological hallmarks of Alzheimer’s disease (AD), Parkinson’s disease (PD), frontotemporal lobar degeneration (FTLD) and amyotrophic lateral sclerosis (ALS) start in pediatric ages in MMC residents, and by the end of the second decade, they have a well-defined quadruple aberrant neural pathology-documented in forensic autopsies (Calderón-Garcidueñas et al., 2022a; Calderón-Garcidueñas et al., 2017; Calderón-Garcidueñas and Ayala, 2022; Calderón-Garcidueñas et al., 2024b), very similar to the pathology overlap seen in 365 University of Kentucky (Karanth et al., 2020) autopsies of unimpaired and cognitively impaired white 83.8 ± 8.8y individuals. The documentation of AD, PD, FTLD, and ALS in MMC subjects is not a surprise (Calderón-Garcidueñas et al., 2022a; Calderón-Garcidueñas et al., 2017; Calderón-Garcidueñas and Ayala, 2022; Calderón-Garcidueñas et al., 2022b; Calderón-Garcidueñas et al., 2024b; Calderón-Garcidueñas et al., 2018a), given the strong association between neurodegenerative diseases and air pollution exposures across the world (Shi et al., 2023; Kritikos et al., 2022; Rajendran et al., 2022; Alemany et al., 2021; Rhew et al., 2021; Baranyi et al., 2022; Parra et al., 2022; Jung et al., 2015; Lee P. C. et al., 2016; Chen et al., 2017; Shi et al., 2021). Transmission electron microscopy (TEM) and energy-dispersive X-ray spectrometry (EDX) have shown Fe, Ti, Hg, W, Al, and Zn spherical and acicular NPs in key noradrenergic and dopaminergic nuclei and cerebellum in MMC children and young adults, as well as Fe, Ti, and Al alloys and Hg NPs in fetal brains and placentas (Calderón-Garcidueñas et al., 2022a; Calderón-Garcidueñas et al., 2022b; Calderón-Garcidueñas et al., 2024b; Calderón-Garcidueñas et al., 2020).

Given that targeted brain structures for AD, PD, FTLD and ALS exhibited UFPM/NPs single domain accumulation in specific organelles (Calderón-Garcidueñas et al., 2022a; Calderón-Garcidueñas et al., 2017; Calderón-Garcidueñas and Ayala, 2022; Calderón-Garcidueñas et al., 2022b; Calderón-Garcidueñas et al., 2024b; Calderón-Garcidueñas et al., 2020), and that young MMC adults are showing significant MRI frontal, temporal, caudate and cerebellar atrophy, hand in hand with low Montreal Cognitive Assessment (MoCA) total and index scores (Calderón-Garcidueñas et al., 2022c), our aim for this work was to measure magnetic particle variables, including saturation isothermal remanent magnetization (SIRM)-a bioindicator for monitoring magnetic anthropogenic air pollutants (Aguilar-Castillo, 2023; Kardel et al., 2023; Adachi and Buseck, 2010; Bautista-Hernández et al., 2023; Aguilera et al., 2021; Bautista et al., 2014; Delgado et al., 2019; Cejudo et al., 2022; Lee P. K. et al., 2016), together with *in situ* identification of nanosized metals, metalloids and natural elements and their motion behavior under low magnetic fields from 202 brain samples showing early AD, PD, FTLD and ALS neuropathology and a cohort of ALS cases and their controls.

Magnetic methods are reliable and powerful techniques for identification of the relative contribution of anthropogenic, industrial magnetic pollutants conducive to environmental preventable factors for common neurodegenerative diseases affecting millions of people around the world.

Brain magnetic particles in subcellular structures are ubiquitous in young urban residents and sensitive to low magnetic fields, with critical motion behaviors posing a high organelle and cell membranes damage risk in targeted brain regions of utmost importance for the early pediatric development of Alzheimer and Parkinson’s diseases, Frontotemporal lobar degeneration and amyotrophic lateral sclerosis.

## 2 Materials and methods

### 2.1 Study cities and air quality

**Figure 1:**
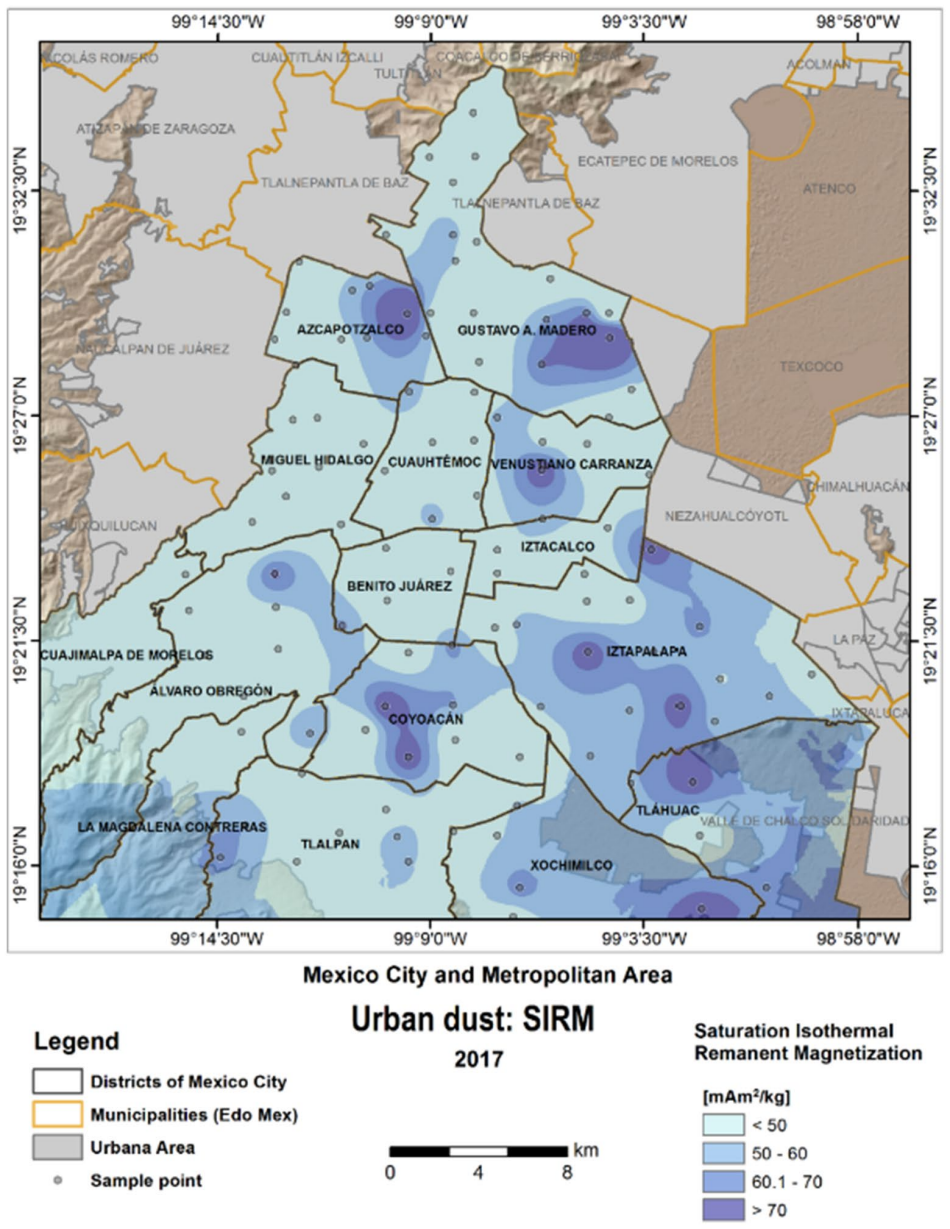
Saturation isothermal remanent magnetization SIRM values were obtained from dust urban road samples in MMC in March 2017. Darker blue color indicates areas with high concentration of magnetic minerals (ferrimagnetic). A highly populated area in MMC Iztapalapa (~2 million people) is a prime example of high ferrimagnetic materials.

Metropolitan Mexico City (MMC) has 22 million residents who have been chronically exposed to high concentrations of PM_2.5_ and NPs for the last 3 decades (Aguilar-Castillo, 2023; Velasco et al., 2019; Caudillo et al., 2020; Dunn et al., 2004; Kleinman et al., 2009). In Figure 1, the distribution of values of SIRM -an indicator of ferrimagnetic minerals in road dust- shows the significant MMC concentrations’ variation for 2017.

Heavy metal concentrations have been measured from the street dust in MMC by our group, and the methodology included digestion of dust samples and analysis in triplicate with an Agilent Technologies 5100 Inductively Coupled Plasma Optical Spectrometer (ICPOES) (US: EPA method 6010C) (Bautista-Hernández et al., 2023; Aguilera et al., 2021; Bautista et al., 2014; Delgado et al., 2019; Cejudo et al., 2022). Studies have shown Spearman correlation coefficients and principal component analysis (PCA) associations between Pb and Cr. Heavy metals in street dust within MMC correlates with industrial, vehicular and street yellow paint sources (Lee P. K. et al., 2016). The metals with the highest concentrations across MMC are Cr, Zn, Cu and Pb. Moreover, three major industrial and high vehicular areas within MMC: Cuauhtémoc, Venustiano Carranza and Gustavo A. Madero are the worst in terms of heavy metals in urban dust (Bautista-Hernández et al., 2023; Aguilera et al., 2021; Bautista et al., 2014; Delgado et al., 2019; Cejudo et al., 2022). Remarkably, high transit avenues close to the International Mexico City Airport and northern industrial areas have the highest concentrations of ferrimagnetic particles and three factors contribute to higher concentrations: high traffic vehicular intersections, slow mandatory areas ≤30km/h and traffic speed bumps. Between 2011 and 2018 there has been an increase in street dust concentrations of Cu, Pb, and Zn (Cejudo et al., 2022). Metal concentrations and ferrimagnetic particles in urban streets are evidence of anthropogenic pollution across a city (Bautista-Hernández et al., 2023; Aguilera et al., 2021; Bautista et al., 2014; Delgado et al., 2019; Cejudo et al., 2022).

For this work, the definitions of ultrafine particulate matter (UFPM) and industrial nanoparticles (NPs) are interchangeable: particles smaller than 0.1 μm (100 nm) in diameter, commonly referred as PM_0.1_, either from anthropogenic origin, natural sources (volcanic) or industrial origin (i.e., food, electronic waste). This fraction is not measured in MMC.

### 2.2 Study design and brain samples

Two-hundred and three brain samples from forensic MMC autopsies (20/64/2003), ALS and their controls (Dartmouth Health STUDY00028053, CPHS# 28053), and eight samples including olfactory bulb, vagus nerve, hypophysis and optic chiasm from three 11-month-old pigs [IACUC #2021/026] are included in the magnetic studies (Supplementary Table S1). The MMC autopsy cases included 14 children (≤18y) with an average age of 9.23 ± 7.73y and 27 adults age 39.8 ± 18.4y.

We took up to 11 different brain regions from selected autopsies, and their selection was based on targeted neuropathology regions (Calderón-Garcidueñas et al., 2022a; Calderón-Garcidueñas et al., 2017; Calderón-Garcidueñas and Ayala, 2022; Calderón-Garcidueñas et al., 2021; Calderón-Garcidueñas et al., 2024b; Calderón-Garcidueñas et al., 2018a; Calderón-Garcidueñas et al., 2020) and the MRI structurally abnormal regions reported in seemingly healthy MMC residents (Calderón-Garcidueñas et al., 2022c). All subjects had unremarkable macro and microscopic examination of extra-neural key organs. Examination of MMC autopsy materials was approved by the Forensic Institute in Mexico City [20/64/2003]. Autopsies were performed 4.1 ± 1.7 h after death and brains were examined macroscopically, brain sections were selected for light and electron microscopy, and frozen tissues collected for magnetic studies.

The age, sex and brain anatomical regions studied in each subject are shown in Supplementary Table S1. Paraffin embedded tissue was sectioned at a thickness of 6 μm and stained with hematoxylin and eosin (HE), while immunohistochemistry (IHC) was performed on serial sections as previously described (Calderón-Garcidueñas et al., 2018a). Antibodies included: β amyloid 17–24, 4G8 (Covance, Emeryville, CA 1: 1,500), PHF-tau8 (Innogenetics, Belgium, AT-8 1:1,000), Phospho-Alpha-synuclein (Ser129) Antibody (PA5-104885) ThermoFisher, Anti-human α-Synuclein mab 5G4, amino acid sequence 44–57 TKEGVVHGVATVAE, Roboscreen GmbH, Lepzig, Germany; 1;1000TDP-43 Polyclonal antibody 10782-2-AP Protein Tech, TDP43 mab2G10, Roboscreen GmbH, Lepzig, Germany 1;1000. Brain tissues were blindly investigated for purposes of AD, PD, and TDP-43 pathology by Board certified neuropathologists and anatomical pathologists. Early subcortical and cortical lesions, the amyloid-β protein phases, brainstem tau pathology, alpha-synuclein and TDP-43 pathology were investigated in this work (Braak et al., 2011; Braak and Del Tredeci, 2015; Alafuzoff et al., 2008; Thal et al., 2002; Rahimi and Kovacs, 2014; Rüb et al., 2016; Del Tredeci and Braak, 2022; Braak et al., 2003; Jellinger, 2022).

### 2.3 Magnetic experiments

We characterized the behavior and concentration of magnetic minerals in samples of brain using various magnetic parameters. Each brain sample was weighed and placed inside 8 cm^3^ cubes and refrigerated at 4°C before beginning the measurements.

The present study analytically examined the acquisition of anhysteretic remanent magnetization (ARM) and saturation isothermal remanent magnetization (SIRM). The natural remanent magnetization (NRM), anhysteretic remanent magnetization (ARM), and isothermal remanent magnetization curves were measured with AGICO JR6, a spinner magnetometer.

ARM is produced by a gradual application of an alternating magnetic field in the presence of a weak steady magnetic field. ARM is strongly sensitive to the presence of small single domain grains (SD), with size varying between 0.03 and 0.06 μm for magnetite grains (Hunt et al., 1995; Dunlop and Özdemir, 1997). Low values of the ARM reveal a weak concentration in magnetic, small, essentially single domain grains (SD) (Sari et al., 2016). Thus, for ARM, an alternating magnetic field (AF) is superimposed on a constant magnetic field H_DC_, so that the total field H (t) is given by:


$$H\left(t\right)=H_{AF}\left(t\right)\sin\left(\omega t\right)+H_{DC}$$

Where, *t* is the time, ω the angular frequency and *H_AF_* (*t*) the amplitude of the alternating field (Vasquez et al., 2018).

We acquired ARM in a peak AF-field of 100 mT and bias fields of 30, 40, and 50 μT, using a LDA5 AF demagnetizer equipped with PAM1 unit. ARM susceptibility was calculated by dividing the mass-specific ARM by the size of the biasing field (0.05 mT).

The *isothermal remanent magnetization* (*IRM*) is the remanence left in the sample after a permanent field (1–1,000 mT) has been applied for a short time (1 s). It is acquired without changes in temperature, and it is related to the coercivity sample spectrum and can thus be used for identification of magnetic minerology (i.e., magnetite is typically saturated at 300–500 mT) and concentrations (Evans and Heller, 2013; Li et al., 2017; Ma et al., 2023; Narayanaswamy et al., 2022; Mohapatra et al., 2013). IRM acquisition curves were performed in all samples; each sample was subjected to a continuing increase magnetic field in the same direction. A pulse magnetizer IM-10 (ASC Scientific) provided direct fields up to 1,000 mT and backward field of 300 mT (opposite direction). The IRM acquired at 1,000 mT was considered the saturation isothermal remanent magnetization (SIRM). IRM acquisition and demagnetization curves are also useful for studying the effects of interactions between magnetic particles. ARM and SIRM values were normalized to mass for the analyses.

The S-ration -useful to estimate the presence of soft magnetic mineral with low coercivity, such as magnetic fine particles (>0.05 μm), essentially single domain (SD) magnetite-was calculated as S_300_ = IRM_−300_/SIRM, in this case we used IRM 300 mT (IRM_300_) in a backward field.

### 2.4 Scanning microscopy and energy-dispersive X-ray spectrometry

Scanning Electron Microscopy was carried out using a field emission ultra-high resolution Scanning Electron Microscope JEOL- JSM-7800F equipped with Oxford Instruments EDS detector and AZtec 2.2 software. For identification and elemental analysis of UFPM/NPs, we cut 70 nm unstained tissue sections and placed them on Ni and/or Au mesh grids. The TEM grids were placed upon a graphite sample holder and the backscattered electron detector was used to perform a phase contrast study of the particle size and morphology. Elemental chemical analysis was performed using the EDS detector, which consists of the detection of X-rays produced by the interaction of the electron beam with the sample. Chemical mapping was done to study the distribution of elements present in the sample, using the X-Max^n^ energy-EDX detector from Oxford Instruments.

### 2.5 Transmission electron microscopy

TEM Studies were performed using three mm^3^ block brain samples. Samples were cut with ceramic knives and handled with plastic forceps, free from metal contamination. Sections were fixed in 2% paraformaldehyde and 2% glutaraldehyde in sodium phosphate buffer for TEM and EDX studies. Brain samples were embedded in EPON. Semi-thin sections 0.5 to 1μm were cut and stained with toluidine blue for light microscopy. Sections were examined under a Carl Zeiss Axioskop 2 PLUS microscope equipped with an AxioVision REL 4.8 imaging system. We selected the electron microscopy areas from the toluidine blue sections. Ultrathin sections 60–90 nm were cut and collected on grids covered with Formvar membranes. Sections were examined with a JEM-1011 (Japan) microscope. Each EM picture was evaluated separately, and we captured ultrastructural images of vascular and neural elements, including subcellular organelles. The focus of the brain evaluation using conventional TEM (JEOL-1011, Osaka, Japan, operated at 80 kV) was to document the integrity of the neurovascular unit (NVU), define the location of the electrodense UFPM/NPs and describe the structural changes in cell organelles of different cell types.

We analyzed all samples blind to case and grids/tissue sections and grid areas were randomly selected and methodically scanned.

## 3 Statistical analysis

Our sample size of 203 brain samples was defined *a priori* by sampling logistics in the 5y study period and focused on mostly residents in MMC. We concentrated on summary statistics and summary of the targeted magnetic variables. We compared magnetic variables between the different cohorts, particularly the differences in subcortical versus cortical regions across age groups and the motor areas in MMC cases versus ALS and ALS controls. We performed the statistical analyses using Excel and the statistical software “R” (http://www.r-project.org/).

## 4 Results

### 4.1 Air pollution

Metropolitan Mexico City (MMC) is a prime example of uncontrolled urban growth and unsuccessfully controlled environmental pollution for the last 27 years (Aguilar-Castillo, 2023; Velasco et al., 2019; Caudillo et al., 2020; Dunn et al., 2004; Kleinman et al., 2009). Our study included MMC children and adults, young pigs kept in the outdoor/indoor facility of the Instituto Nacional de Pediatría in SWMMC and brain samples from US Veterans with ALS and their age-matched controls.

**Figure 2:**
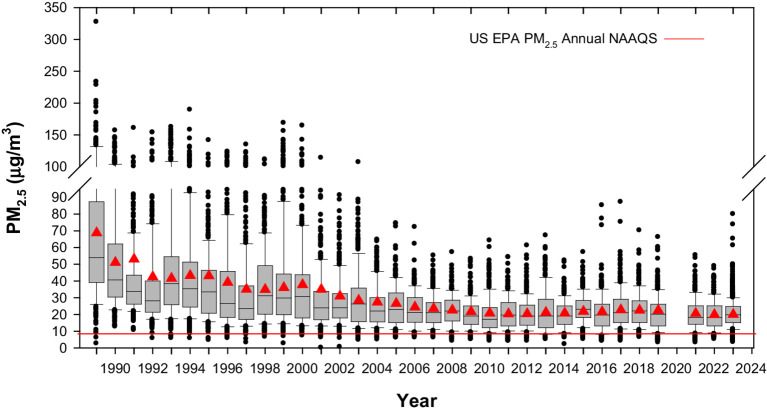
Trend of annual averaged over 3 years box plots of mean 24-h PM_2.5_ for five representative MMC monitoring stations from 1989 to 2023. COVID period information is not available. The red triangles represent the annual mean and the red line the current PM_2.5_ annual US EPA NAAQS (9 μg/m^3^). Box plots from the years before 2004 were estimated from available information of PM_10_ 24-h averages since 1989 and the mean slope of the correlation PM_10_ vs. PM_2.5_ between 2004 and 2007. Data from: http://www.aire.cdmx.gob.mx/default.php#.

**Figure 3:**
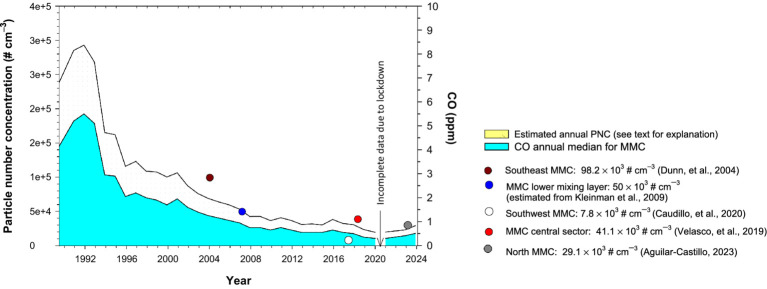
Trends of estimated particle number concentrations (PNCs)-a calculated estimation of nanoparticles- and the associated annual medians of 1-h average Carbon monoxide (CO) for five representative monitoring stations of the MMC from 1989 to 2023. The colored circles in the figure correspond to the medians of PNCs measured by the authors referenced in Aguilar-Castillo (2023), Velasco et al. (2019), Caudillo et al. (2020), Dunn et al. (2004), and Kleinman et al. (2009). CO data source: http://www.aire.cdmx.gob.mx/default.php#.

The MMC area is over 2,000 km^2^ and lies at an elevated basin 2,200 m above sea level. MMC has 22 million people, over 50,000 industries, and 5 million vehicles consuming more than 50 million liters of petroleum fuels per day. Motor vehicles in MMC, including diesel heavy vehicles, release abundant amounts of primary PM_2.5_, elemental carbon, particle-bound polycyclic aromatic hydrocarbons, carbon monoxide, nitrogen oxides and a wide range of toxins and other toxics, including lipopolysaccharides, formaldehyde, acetaldehyde, benzene, toluene, and xylenes (Velasco et al., 2019; Caudillo et al., 2020; Dunn et al., 2004). MMC subjects in this study have been exposed to significant concentrations of PM_2.5_ above the current US EPA annual standard (9 μg/m^3^) (Figure 2) and ultrafine PM and nanoparticles (NPs) (Figure 3) (Aguilar-Castillo, 2023; Velasco et al., 2019; Caudillo et al., 2020; Dunn et al., 2004; Kleinman et al., 2009).

Metropolitan Mexico City residents have been continually exposed to PM_2.5_ above 9 μg/m^3^ for the last 27 years and to nanoparticles.

### 4.2 Brain magnetic studies

#### 4.2.1 Anhysteretic remanent magnetization

**Figure 4:**
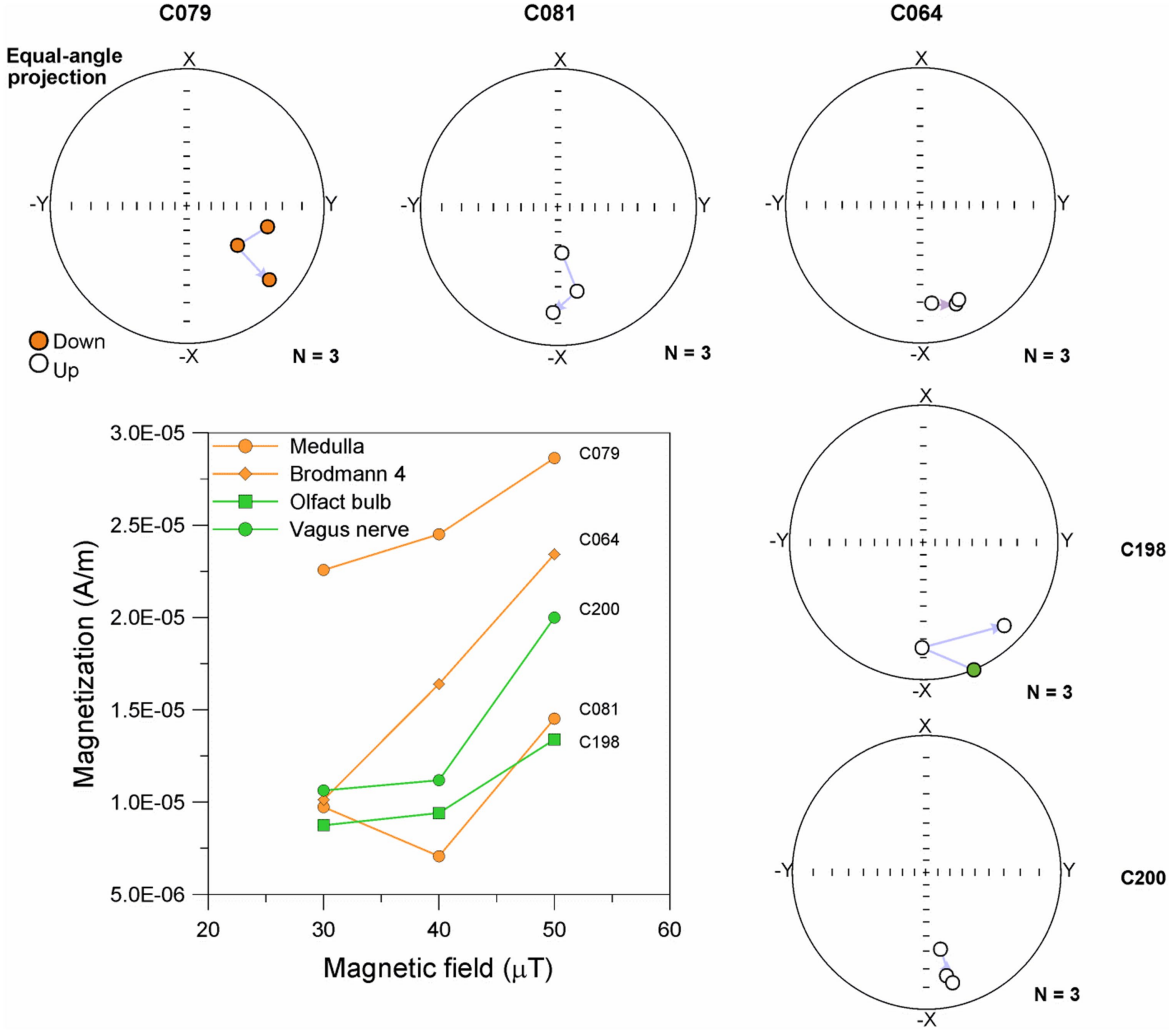
Curves of ARM obtained in an AC field of 100 mT and DC field of 30, 40, and 50 μT and equal-angle projection of magnetization direction of brain samples.

ARM is a magnetization that magnetic particles acquire when subjected to an alternating field (AF) of gradually decreasing amplitude (*H_AF_*) with a constant decrement (Δ*H_AF_*/cycle) simultaneously with a steady, unidirectional DC field (*H_DC_*). The ARM is measured when both AF and DC fields are zero. The ARM curves are produced by gradually reducing a strong alternating magnetic field in the presence of a weak steady magnetic field. ARM curves of the different brain anatomical regions showed magnetization increase between 30 and 50 μT-an indicator of the presence of ferromagnetic particles pseudo-single domain (PSD) or single domain (SD) (particle size <76 nm) (Li et al., 2017) (Figure 4).

In Figure 4, C079 (Medulla) in a 79y old ALS patient exhibited a proportional increase in ARM (22.6 × 10^−6^ A/m to 28.6 × 10^−6^ A/m) between 30 and 50 μT with a ratio of 0.3 ×10^−6^ A/m per μT. Moreover, a magnetic vector change in direction (4–27°) was observed when the sample was exposed to both AC and DC magnetic fields. In contrast, sample C081 (Medulla) in a 68y old male with a diagnosis of both ALS and FTLD, did not show a proportional increase of the ARM between 30 and 50 μT. The ARM value decreased by 27% with the application of an AC and DC magnetic field of 40 μT, while for the DC magnetic field of 50 μT, the value increased by 50% from the initial value (9.7 × 10^−6^ A/m). Variation in magnetic vector direction ranging from 6^°^ to 44^°^ was observed with each AC and DC magnetic field applied.

On the other hand, sample C064 (Brodmann 4) in a 74y old male control (i.e., no ALS disease) show a proportional increase of the ARM (10.1 × 10^−6^ A/m to 23.4 × 10^−6^ A/m) between 30 and 50 μT with a ratio of 0.6 × 10^−6^ A/m. Moreover, a magnetic vector change in direction (2–15^°^) was observed when the sample was exposed to both AC and DC magnetic fields.

Remarkably, the 79y old ALS (C079) sample had a brisk magnetic vector direction response compared to the 68y old (C081). The mineral magnetic contained in the medullary ALS samples C079 (ARM: 1.04 μAm^2^/kg) and C081 (ARM: 0.97 μAm^2^/kg) exhibited a change in the direction of the magnetic vector (between 4^°^ and 27^°^) and increases in ARM intensity when exposed to the AC and DC fields. While the C064 Brodman 4 Control sample from a 74y old male ALS control had minimal variation in magnetic vector direction and an acquisition of ARM greater than samples Medulla. These observations may suggest distinct magnetic minerals profile may be subjected to significant movement under the influence of AC and /or AD magnetic fields in ALS vs. controls.

Samples C198 (Olfactory bulb) showed an increase in ARM (8.7 × 10^−6^ A/m to 13.4 × 10^−6^ A/m), between 40 and 50 μT the increase of ARM showed a ratio of 0.4 per μT. Moreover, a magnetic vector change in direction (6–45°) was observed when the sample was exposed to both AC and DC magnetic fields. In contrast, sample C200 (Vagus nerve), did not show a proportional increase of the ARM between 30 and 50 μT. The ARM increased by 42% with the application of an AC and DC magnetic field of 40–50 μT. Variation in magnetic vector direction ranging from 1° to 20° was observed with each AC and DC magnetic field applied.

The magnetic mineral in the olfactory bulb C198 exhibited a significant variation in magnetic direction (greater than 40°) when exposed to AC (100 mT) and DC (40 μT) fields. The magnetic mineral in Brodmann 4 C064 and vagus nerve C198 samples exhibited little variation, less than 15 degrees in magnetic direction when exposed to AC and DC fields and the intensity of ARM increased between 40 and 50 μT, indicating that the magnetic mineral had a subtle change in position during exposure to the magnetic field.

#### 4.2.2 Curves of isothermal remanent magnetization and saturation isothermal remanent magnetization

We obtained the curves of IRM and values of SIRM in 202 fresh-frozen brain samples (Supplementary Table S1). The SIRM captures the magnetic contribution of ferrimagnetic particles ~ 20 nm as to be magnetically unstable at room temperature interacting with the presence of the magnetic field. All 11 selected brain regions exhibited the presence of magnetic particles during IRM_1000_ (SIRM) acquisition (Table 1). T1, T2, and T3 magnetic motion behavior induced by applied magnetic fields of 25–1,000 mT AC was defined as follows: T1 no particle movement, T2 small displacement of the magnetic particle, T3 significant variations in the direction of the magnetic vector during the acquisition and inverse acquisition of IRM, with variations ≥90 degrees. Remarkably, the motion behavior of UFPM/NPs is strikingly different according to brain regions for each individual.

**Table 1:** Two-hundred and three samples from 11 different brain regions with SIRM measurements.

	Average age ± SD	Brain samples g	Number of samples	ARM _50_ μAm^2^/kg	SIRM μAm^2^/kg	IRM_300_ μAm^2^/kg	S_300_ AD
All samples	35.94 ± 25.0	0.6193	203	0.6430	34.94	35.50	0.9708
Cortical samples	41.83 ± 24.4	0.7457	81	0.2790	20.02	20.92	0.9728
Subcortical samples	33.83 ± 24.3	0.5430	154	0.9145	45.93	46.38	0.9693
T1 motion behavior	35.21 ± 24.0	NA	174	0.5621	28.82	28.26	0.9856
T2 motion behavior	43.06 ± 32.3	NA	18	0.8756	62.11	68.90	0.8833
T3 motion behavior	33.18 ± 24.9	NA	11	1.5409	86.86	94.79	0.8655
Brodmann 4	58.5 ± 24.6	0.97	46	0.1363	11.25	10.67	0.9625
Caudate	29.0 ± 17.8	0.37	17	2.85	97.62	106.51	0.9895
Putamen	32.1 ± 19	0.67	18	0.3906	25.65	25.16	0.9765
Cerebellum	27.5 ± 18.5	0.81	22	0.2105	17.21	16.87	0.9864
Hippocampus	32.2 ± 31.4	0.56	7	0.338	34.54	29.95	0.9200
Cingulate anterior	38.5 ± 19.4	0.77	13	0.1362	10.53	9.97	0.9615
Thalamus	24.0 ± 8.4	0.63	8	0.8863	57.75	84.38	0.9750
Temporal	35.1 ± 21.0	0.85	29	0.2161	18.31	20.40	0.9750
Olfactory bulb	1.00	0.50	4	0.2100	16.28	15.00	0.9500
Vagus + Optic chiasm	1.00	0.20	2	1.0500	74.12	71.50	1.0000
Hypophysis	1.00	0.10	2	0.8100	42.35	42.11	1.0000

The average age of the individuals in the study was 35.9 ± 25.0y, including 56 samples from pediatric ages (≤18y). The youngest MMC resident was an 11m old.

**Figure 5:**
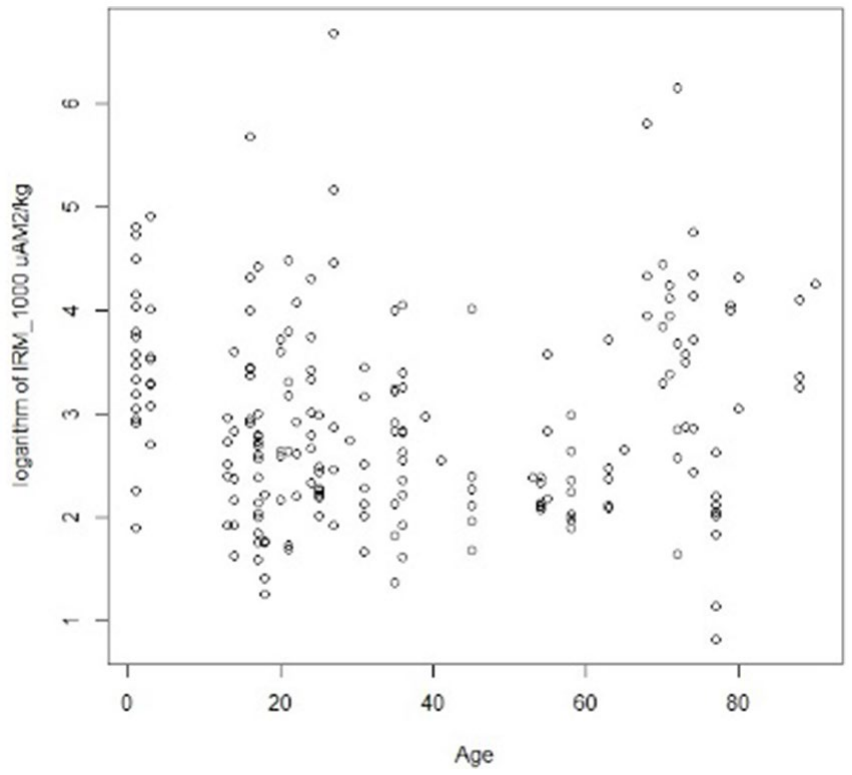
Distribution of highly magnetic particles in the 203 brain samples, including cortical and subcortical regions. Please notice children and young adults exhibit high magnetic particle concentrations.

The distribution of highly magnetic particles IRM_1000_ μAm^2^/kg (SIRM) across all ages, is seen in Figure 5.

**Figure 6:**
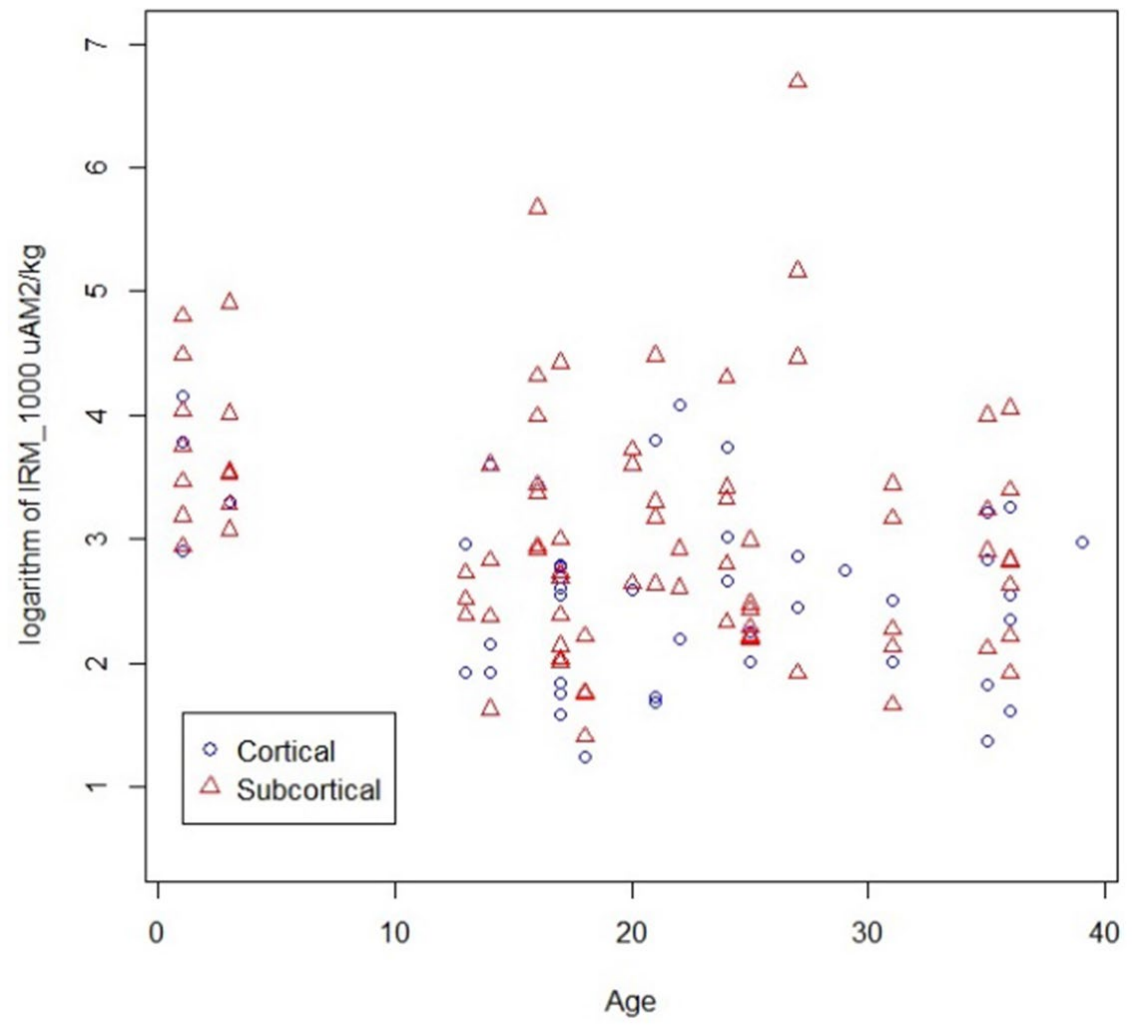
IRM_1000_ (SIRM) measurements in subcortical vs. cortical regions in individuals younger than 40y of age, *p* = 0.0234 higher in subcortical regions.

A significant difference was recorded between the presence of magnetic particles in subcortical versus cortical regions across age groups, i.e., subjects ≤40y, displaying a significant difference in SIRM with higher subcortical versus cortical locations *p* = 0.0234 (Figure 6). No differences in SIRM were recorded in cortical vs. subcortical regions in ≥41y olds *p* = 0.0755.

#### 4.2.3 Brain samples SIRM

**Figure 7:**
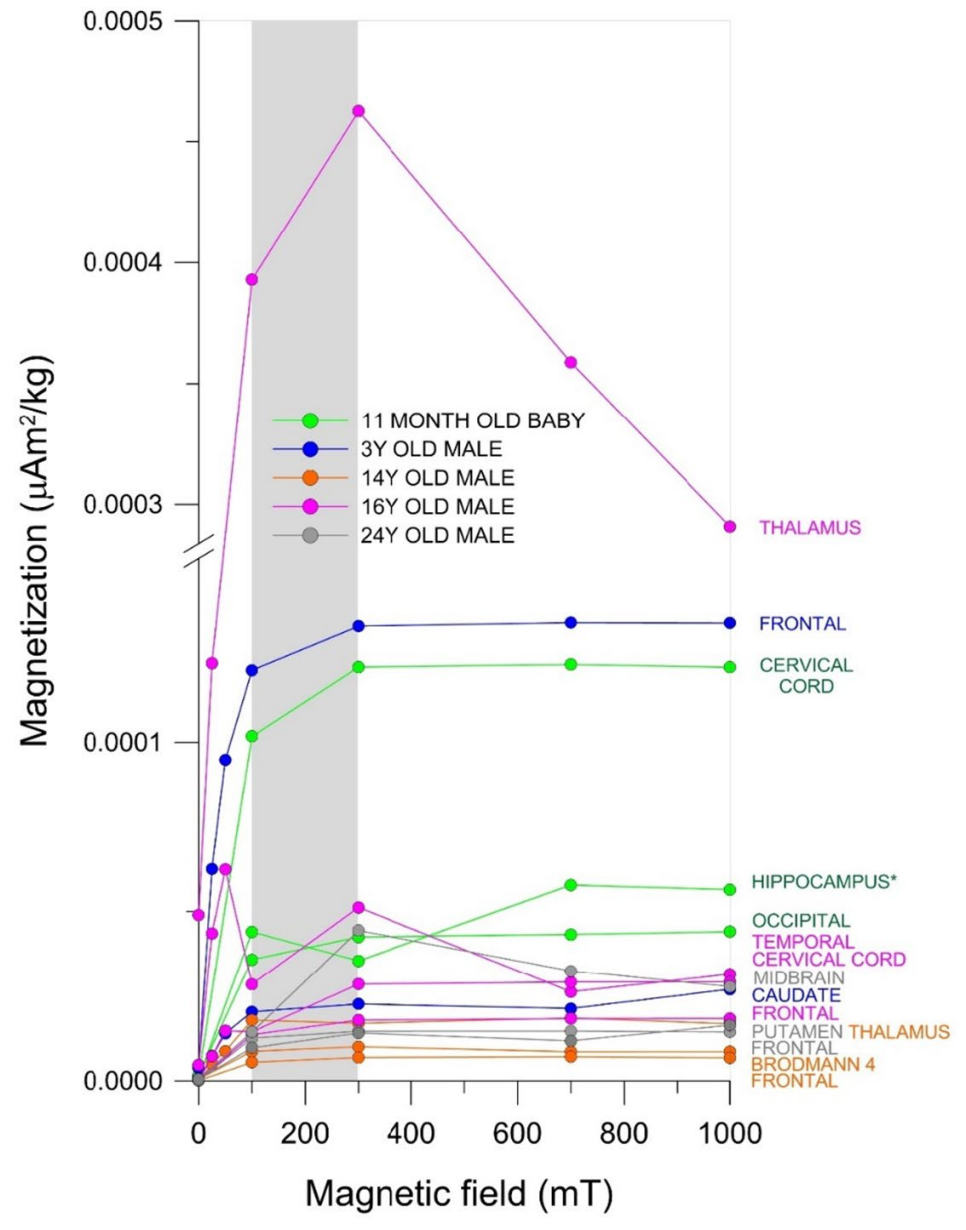
IRM acquisition curves from different brain anatomical areas in males ages 11 months to 24 years. The gray area indicates the mT intensity field at which the ferromagnetic minerals reached IRM saturation values (i.e., magnetization of saturation).

We documented significant differences in SIRM in different brain regions from the same subject. Variations in SIRM were detected, with values between 2.3 and 801.0 μAm^2^/kg. The IRM curves reached saturation upon exposure to magnetic fields 100–300 mT, indicating the presence of magnetic minerals with low coercivity. Interestingly, children samples showed a striking variation in the IRM acquisition curves between 50 and 300 mT, with a significant decrease in IRM values, interpreted as a change in particle location. This behavior was exemplified in the thalamus, temporal and hippocampus of a 16-year-old male and an 11month-old baby (Figure 7).

The decrement in IRM values in curves of acquisition is associated with change of orientation and/or position of the magnetic mineral within the samples as seen in the 16y old thalamus sample (Figure 7). The thalamus, frontal, and caudate samples from children ages 11m, 3y, and 16y showed significant differences in ferrimagnetic material concentrations and the IRM values at 300 mT indicate the thalamic teen sample contained 70% more magnetic material than his frontal sample and 76% more than the 11m old baby caudate sample. The 16y old with Ptau and Aβ_1-42_ in cortical and brainstem locations, α-synuclein in olfactory bulb, and TDP-43 in brainstem, exhibits an overlap of quadruple aberrant AD, PD, and TDP-43 and significant variations in the concentrations of ferrimagnetic material across his brain samples (Figure 7; Supplementary Table S2).

#### 4.2.4 Brain samples magnetic components in equi-angular projection diagrams

**Figure 8:**
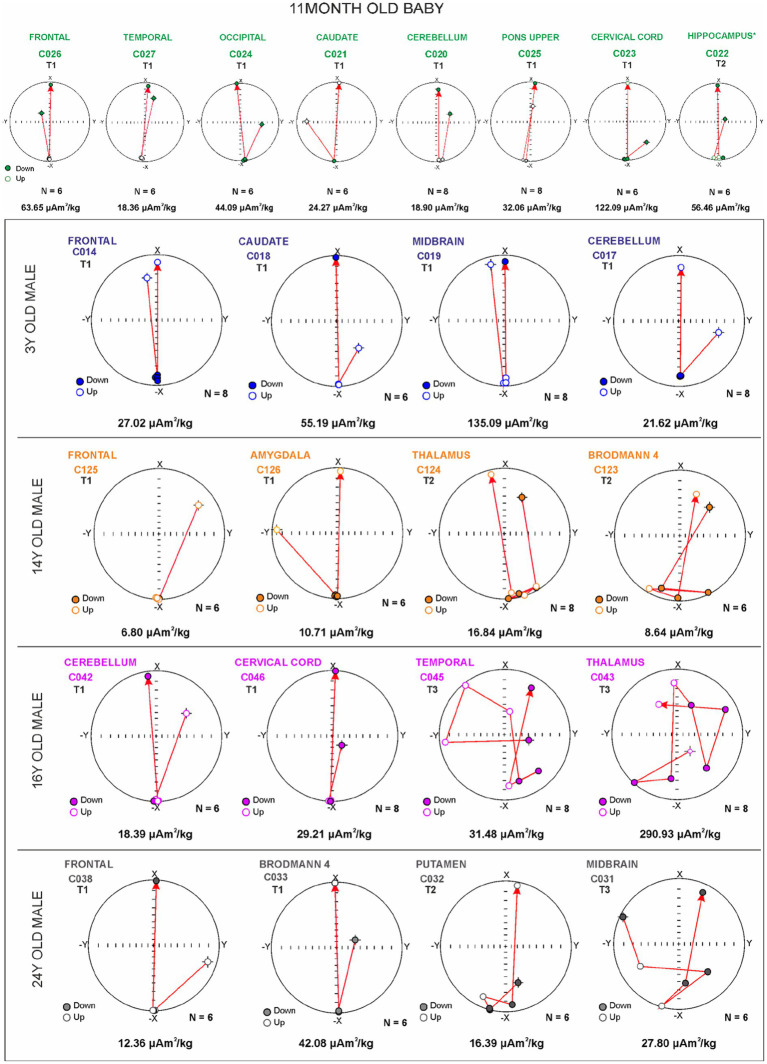
Equi-angular projection diagrams of magnetic vector components of different brain regions in an 11-month-old **(A)** and subjects ages 3, 14, 16, and 24 years **(B)**. T1, T2, and T3 magnetic behavior is correlated with the displacement of magnetic particles, i.e., x in direction to 0 degrees, −x in direction to 180 grades.

An analysis of magnetic components in equi-angular projection diagrams during acquisition of the IRM curves provides evidence of magnetic particle changes in orientation within brain samples (Figure 8). All samples were exposed to a pulse of magnetic field increasing from 25 mT to 1,000 mT. A stable magnetic vector direction should be observed for ferrimagnetic materials, while a change in orientation (180°) is expected when an inverse field of 300 mT is applied.

The changes in position of the magnetic particles can be categorized into three distinct motion behavior groups: T1 does not change during magnetization, maintaining a −x orientation of 180 degrees during the IRM acquisition. T1 inverts its orientation to +x (0 degrees) when a reverse field (300 mT) is applied. T2 demonstrates changes in magnetic orientation, rotating around 180 degrees during the magnetization acquisition, and reversing its direction toward 0 degrees upon the application of the reverse field (with potential changes in the position of the magnetic material near its original position). T3 exhibits changes in orientation throughout the acquisition process. Acquisition of magnetization was observed upon exposures to 50 mT fields, and this behavior is linked to variations in the magnetic mineral’s position (Figures 8A,B).

In Figure 8A, occipital cortex C024 and cervical cord (motor gray) C023 samples from an 11-month-old baby exhibited stable behavior in the direction of the magnetic vector during IRM acquisition (T1 behavior). However, a variation in direction (14° with respect to 180 degrees) was observed in hippocampus C022 (T2 behavior). The inverse acquisition of IRM showed expected values of 0 degrees.

In Figure 8B, midbrain C019 and frontal C014 from a 3-year-old male showed a stable acquisition of IRM with no significant variations in direction observed (T1 behavior). The inverse acquisition of IRM was close to zero degrees. Brodmann 4 C123 and thalamus C124 from a 14-year-old male showed variations in the direction of the magnetic vector during IRM acquisition, ranging from 6 to 45 degrees indicating T2 behavior. The inverse acquisition of IRM showed a variation of 20 degrees with respect to 0 degrees. The frontal C 125 exhibited T1 behavior.

Cerebellum C042 and cervical cord C046 of a 16-year-old male showed a stable acquisition of IRM with no significant variations in direction observed (T1 behavior). The inverse acquisition of IRM showed expected values of 0 degrees. In sharp contrast, temporal C045 and thalamus C043 exhibited significant variations in the direction of the magnetic vector during the acquisition and inverse acquisition of IRM, with variations over 90 degrees (T3 behavior). We had a 24-year-old male, an avid motorcycle driver, exhibiting all three different behaviors: the frontal C028 exhibited T1, the putamen C032, T2, and the midbrain C031 T3 behavior. He had AD and PD hallmarks (Supplementary Table S2).

#### 4.2.5 Brain and cervical motor areas magnetic behavior

Since we have described TDP-43 pathology in MMC children and young adults (Calderón-Garcidueñas et al., 2022a; Calderón-Garcidueñas et al., 2024a; Calderón-Garcidueñas et al., 2024b; Calderón-Garcidueñas et al., 2020; Calderón-Garcidueñas et al., 2022d) in motor and non-motor areas, we compared magnetic results in MMC motor areas versus ALS cases and found a significant difference in ARM_50_, SIRM, and IRM_300_. The differences were also significant between MMC motor areas and non-ALS controls for SIRM and IRM_300_. The higher ARM_50_ (*p* = 0.003), SIRM (*p* = 0.01) and IRM-_300_ (*p* = 0.008) for ALS cases vs. MMC motor area samples were striking (Table 2).

**Table 2:** Comparison between motor areas in MMC versus ALS and non-ALS control cases.

Brain samples	ARM_50_ μAm^2^/kg	SIRM μAm^2^/kg	IRM_300_ μAm^2^/kg
MMC motor vs. ALS	0.0033	0.0121	0.0089
MMC motor vs. ALS controls	0.0537	0.0449	0.0077

The *p*-values are provided in the table.

MMC young residents exhibit magnetic NPs accumulation in motor areas in pediatric ages and remarkably, TDP-43 pathology is also documented simultaneously in pediatric ages (Calderón-Garcidueñas et al., 2022d).

**Figure 9:**
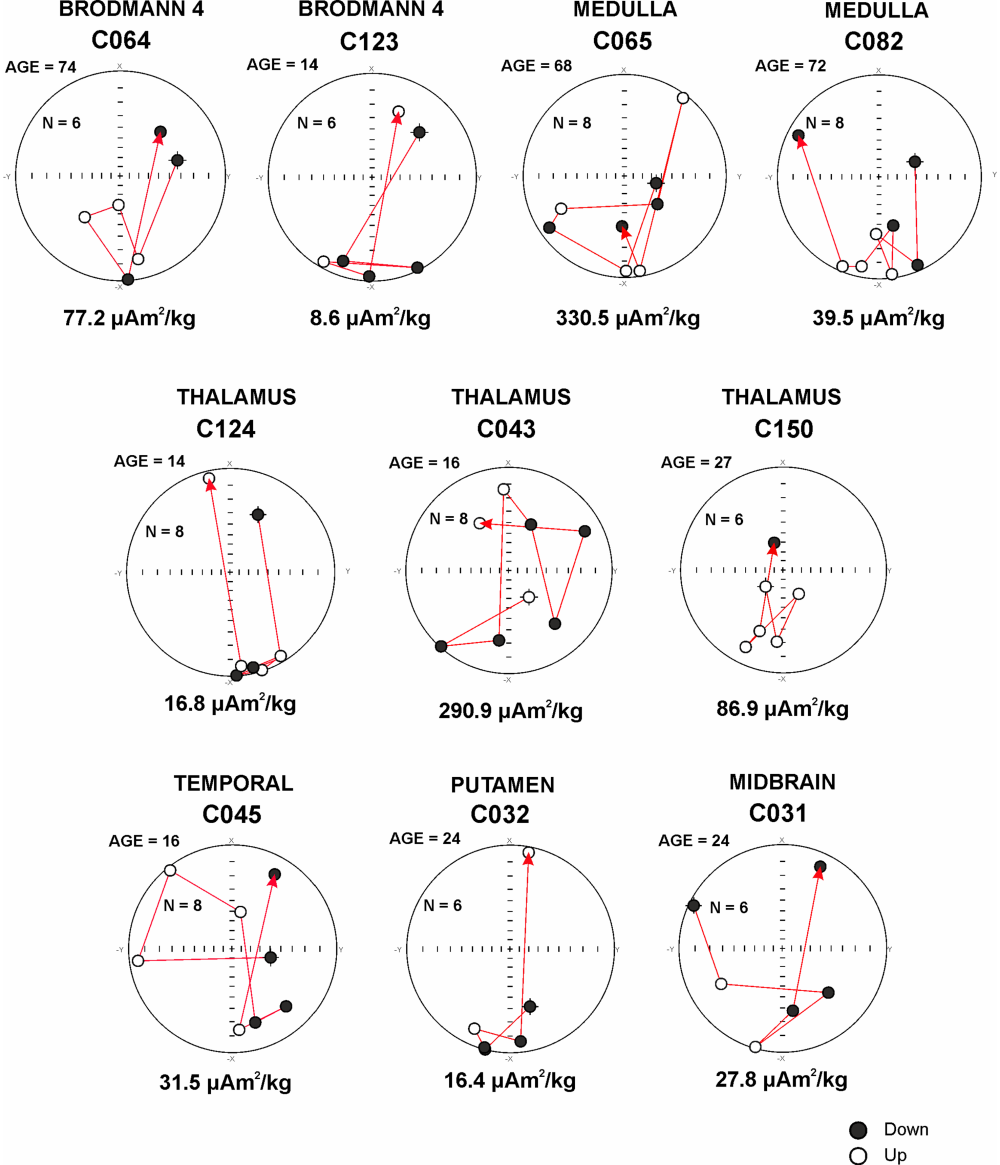
Sensory and Motor areas and T3 behavior in relationship with the MRI acquisition for the targeted region.

Figure 9 shows T3 behavior in samples from Brodmann 4, medulla, thalamus, putamen, temporal and midbrain regions. We documented T3 in teens and elderly subjects regardless of SIRM, thus the highest SIRM value 330 μAm2/kg was documented in 68y male non-ALS medulla and the lowest 8.6 μAm2/kg in a 14y MMC male Brodmann 4 region.

T3 behavior is the result of unstable magnetic NPs with the lowest polydispersity and the highest surface coating molecules; these magnetic particles mostly contain magnetite (Fe3O4), maghemite (γFe2O3), titanomagnetite (Fe3O4-Fe2TiO4) and titanomaghemite (Fe2O3-FeTiO3) (Narayanaswamy et al., 2022; Mohapatra et al., 2013). T3 behavior likely is also impacted by the characteristics of the regional neural tissue, including the presence of organelles capable of accumulating NPs (i.e., lysosomes) and the presence of neurodegenerative changes, both at structural and subcellular levels.

### 4.3 Energy-dispersive X-ray spectrometry

The presence of metal, metalloid, and other elements in UFPM/NPs was verified through EDX. The analysis was aimed at identifying nano size particles elemental composition and documenting the profile and percentage of elements for each particle.

**Figure 10:**
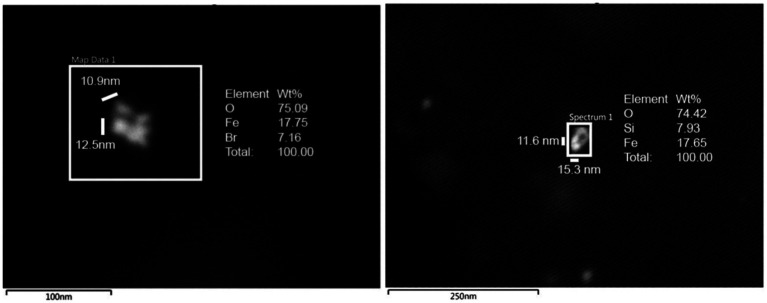
Single domain ≤ 15 nm Fe Br and Si particles in caudate head samples. FeBr_2_ exhibits a strong metamagnetism at 4.2 K and it is a prototypical metamagnetic compound.

**Figure 11:**
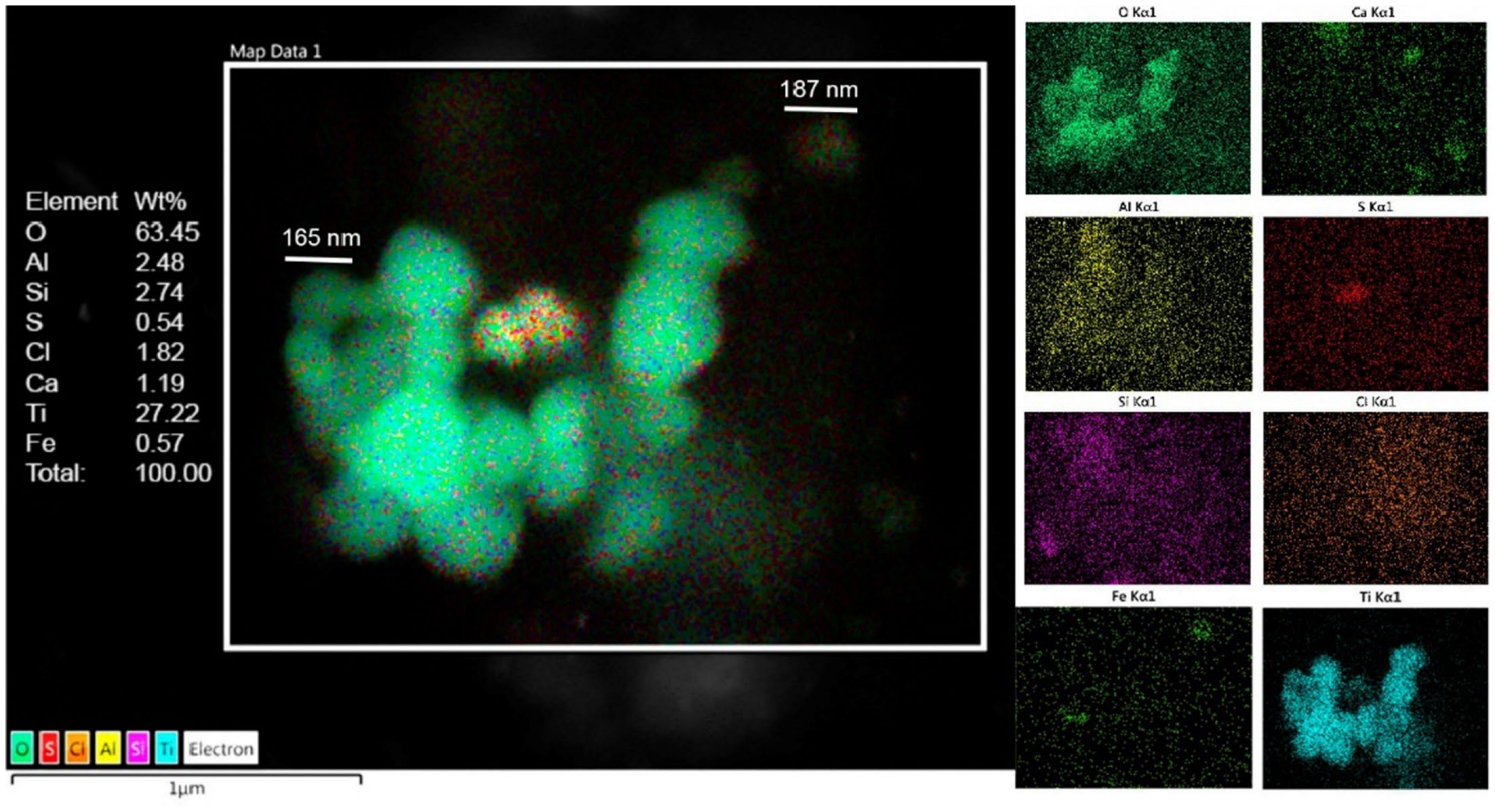
Caudate head sample from a 3y old boy (CO18) displays a combination of Al, Ti, Fe, along Si, S, Ca, and Cl. Oxygen is the major component with 63%.

**Figure 12:**
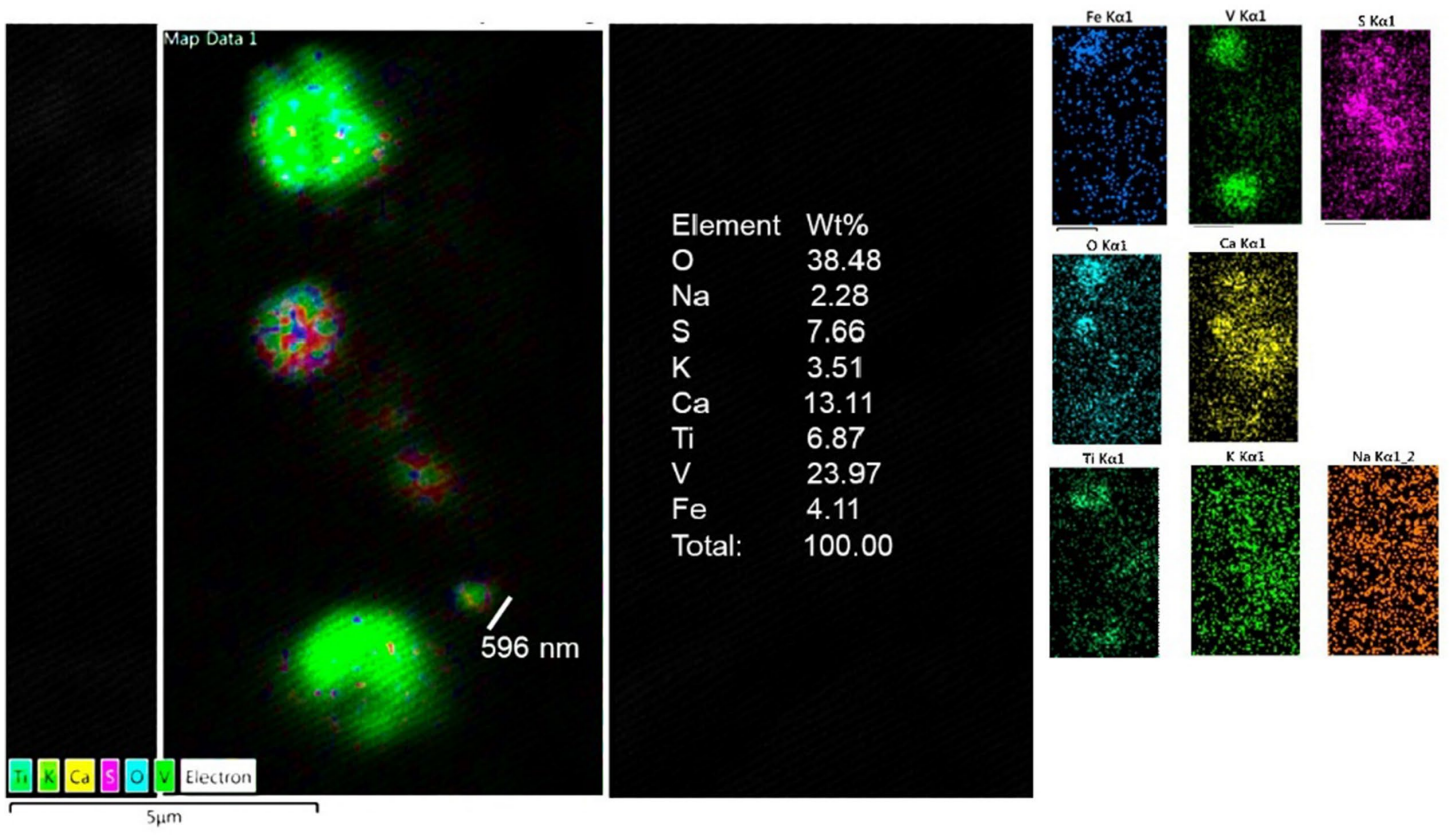
Thalamus from a 16y old boy (CO 43) with a T3 behavior and a high SIRM (Figure 9). Vanadium, titanium and iron were the predominant metals.

**Figure 13:**
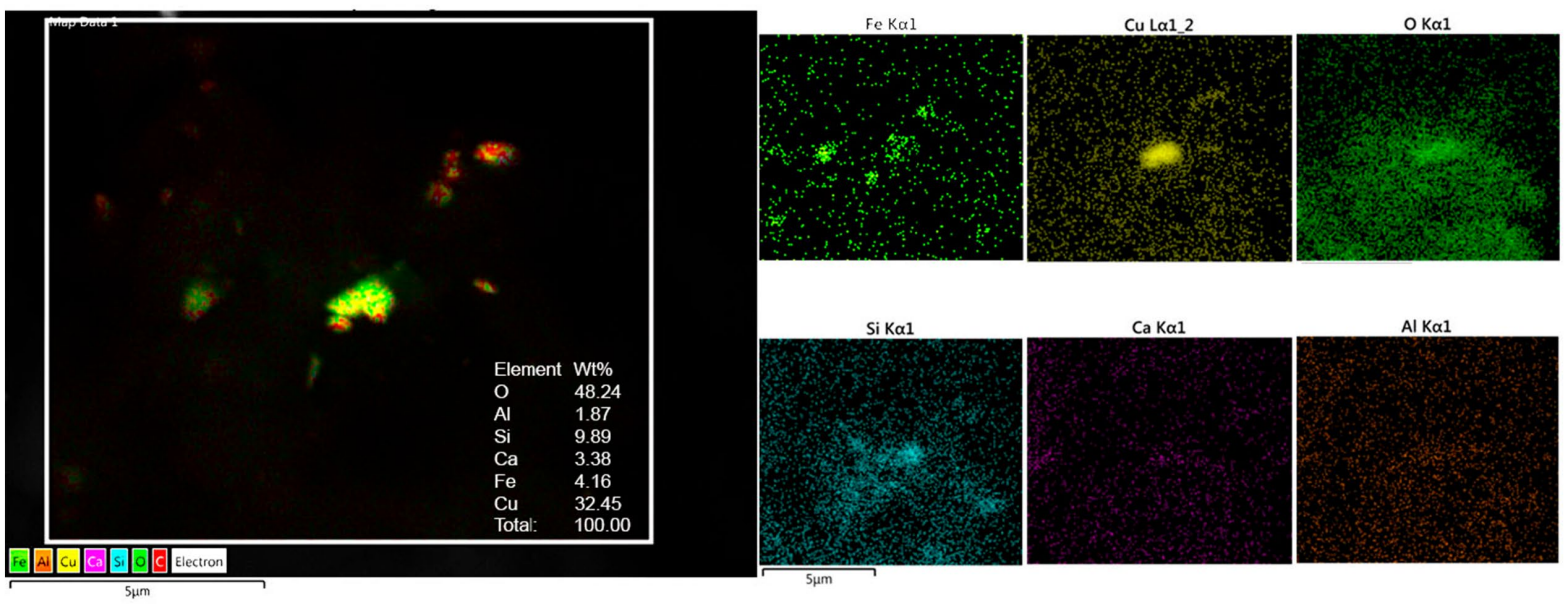
Medulla from 68y old male ALS control (CO65) exhibits a combination of oxidized Cu, Fe, Al, Si, and Ca.

Single-domain, pseudo domain and clusters of Fe, Ni, Co, Ti, V, Hg, Cu, Zn, Cd, Al, Mg, Ag, Ce, La, Pr, W, Ca, Cl, K, Si, S, Na, and Br NPs were identified in frontal cortex, Brodmann 4, hippocampus, thalamus, caudate, putamen, substantia nigra, tectum, periventricular gray, locus coeruleus, cerebellum, olfactory bulb, and vagal nerves (Figures 10–13). Figure 10 illustrates single domain ≤15 nm Fe Br and Fe Si particles in caudate head samples. Metals commonly associated with abrasion and deterioration of automobile catalysts and electronic waste and rare earth elements, i.e., lanthanum, cerium, and praseodymium, are seen in MMC young residents.

## 5 Brain transmission electron microscopy

Brain tissues in this study exhibited the neuropathological hallmarks of AD, PD and TDP-43 associated diseases (Supplementary Table S2). The focus of the TEM evaluation was to document the integrity of the neurovascular unit (NVU) and to define the location of the electrodense NPs in target neural and vascular organelles.

**Figure 14:**
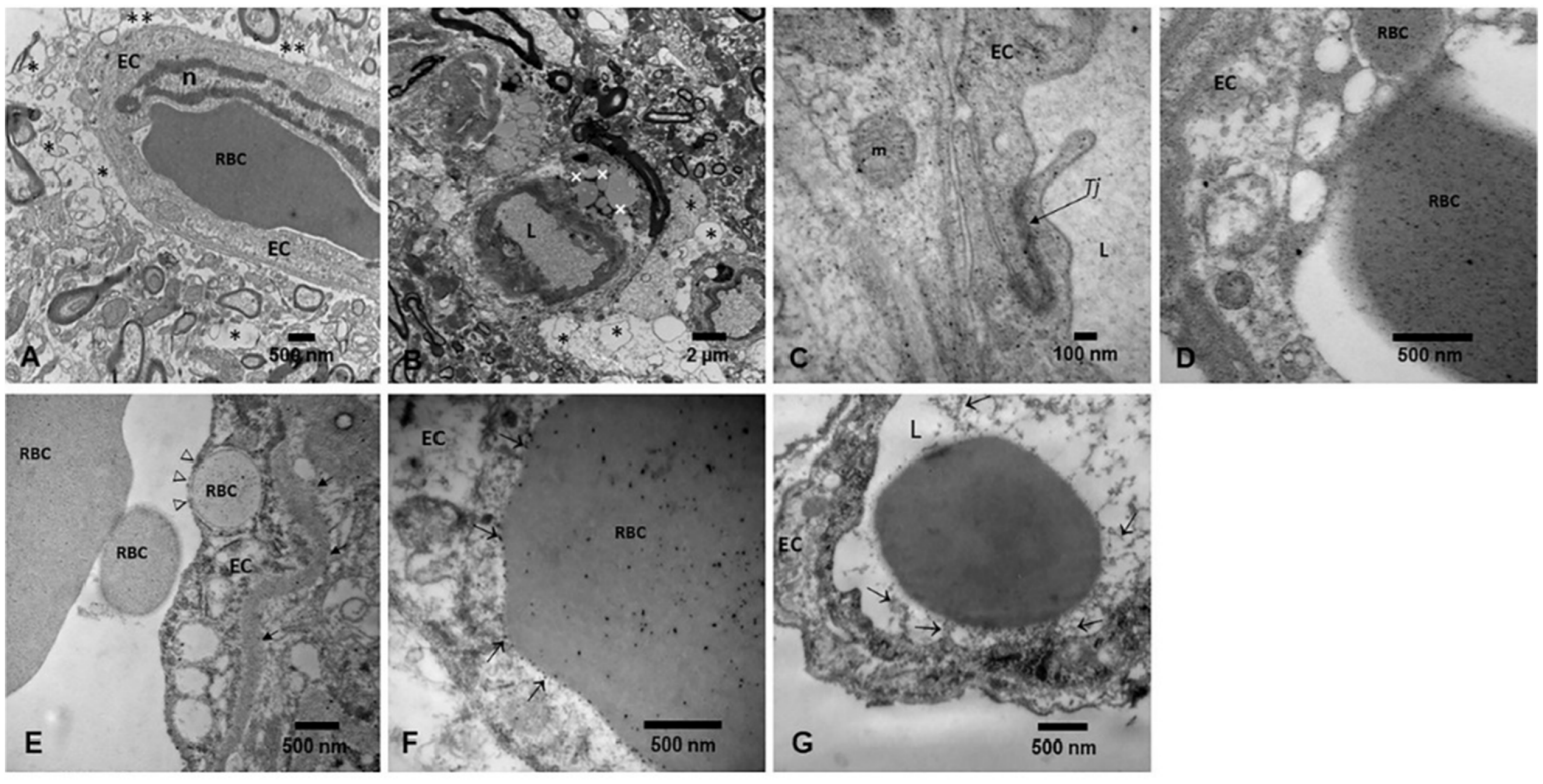
Neurovascular unit pathology in exposed MMC young residents. **(A)** Substantia nigra neurovascular unit with significant perivascular neuropil vacuolization (*), with loss of astrocytic feet to the capillary wall and axonal changes in the vicinity of the blood vessel. A red blood cell is seen in the vessel lumen, in close contact with the endothelial surface. **(B)** Midbrain capillary showing an extensive perivascular neuropil vacuolization damage (*) associated with the accumulation of lipid vacuoles (+) in the proximity of the vessel. **(C)** High magnification of a tight junction cell–cell adhesion complex, between two endothelial cells showing the NPs deposition within the TJ structure (arrow). NPs are also present in the EC cytoplasm and mitochondria (m). **(D)** Olfactory bulb capillary with a luminal RBC loaded with NPs, also present in the EC cytoplasm. An RBC fragment is already within the endothelial cell. **(E)** Seventeen-year-old C087 frontal cortex showing a capillary with a luminal RBC already fragmented and one such RBC fragment already inside the EC (open arrows). **(F)** Cerebellar vermis in the 17y old from **(E)**, the luminal RBC is in close contact with the EC (arrows) and NPs are being transferred inside the endothelium. **(G)** Gray matter cervical spinal cord CO24 toddler showing the activated EC with numerous filopodia (arrows) reaching the RBC surface. A few EC filopodia fragments are free in the lumen. The NVU is an early targeted structure in UFPM/NPs exposures.

**Figure 15:**
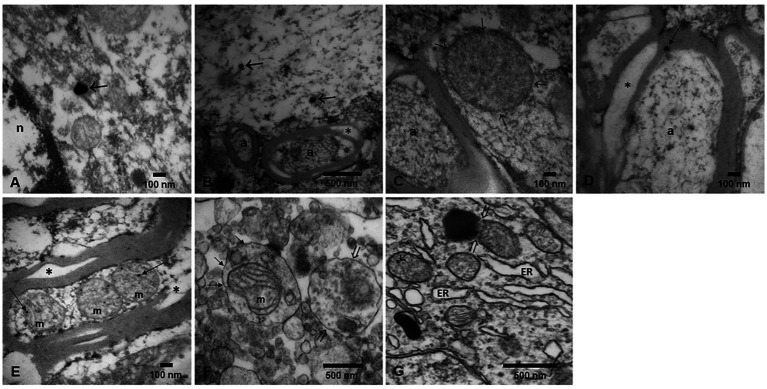
Electron micrographs of NPs in diverse organelles. **(A)** A classical magnetite spherical anthropogenic particle (arrow) is identified in a caudate head neuron, in close proximity to the nucleus. **(B)** Vagus nerve section showing a longitudinally cut axon with two spherical magnetite nanoparticles (arrows). Notice adjacent transverse axons with vacuolated myelin sheets (*). **(C)** Same vagal nerve with a double membrane axonal structure (arrows) showing numerous nanoparticles in the range of 5 nm. The structure could be a mitochondria with no intact cristae. **(D)** A close-up of a vacuolated (*) axonal myelin sheet showing an spherical NPs (arrow). **(E)** Cerebellar vermis with a longitudinally cut axon showing three mitochondria with matrix NPs (arrows). Notice the myelin sheet split (*). **(F)** Caudate neuronal autophagosomes with different stages; abnormal mitochondria (short arrows) are seen along the lysosome fusion and degradation of compartments (open arrows). **(G)** Substantia nigrae dilated endoplasmic reticulum (ER) is identified along mitochondria with NPs (short arrow) and a mitochondria in close contact with neuromelanin containing NPs (open arrows).

Figures 14, 15 illustrate the NVU and organelle pathology associated with the presence of UFPM/NPs. A striking early TEM finding was the transfer of NPs from luminal red blood cells to brain capillary endothelium (EC) and the ongoing erythrophagocytosis along the breakdown of the neurovascular unit illustrated in Figure 14.

TEM showed the extensive organelle pathology and the location of the NPs in critical organelles, including mitochondria, seen in Figure 15.

## 6 Brain light microscopy and immunohistochemistry IHC

**Figure 16:**
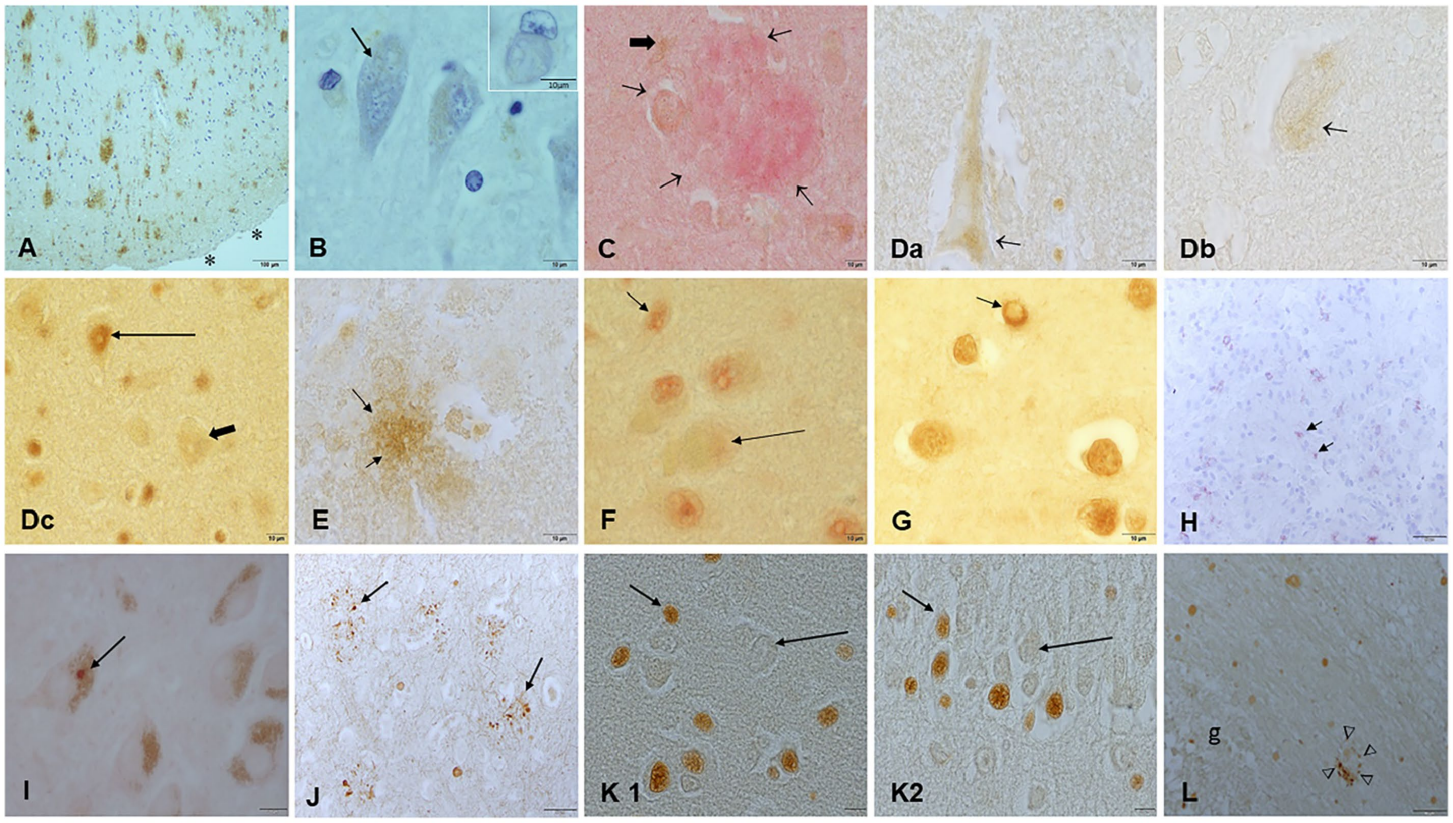
Immunohistochemistry in young MMC residents with hallmarks of AD, PD, and TDP-43 pathology. **(A)** Temporal cortex in a teen residing in an area with high dust SIRM. Amyloid plaques are numerous (brown product) and extend throughout the cortex. The subarachnoid space is marked (*). **(B)** The same child, with numerous Hirano bodies in temporal neurons. Insert shows a ghost neuron with a tangle. **(C)** Thirty-five year old C108 frontal amyloid plaque (red product short arrows) and a reactive astrocyte GFAP+ (black thick arrow). **(Da,Db)** young adults showing p-Tau in frontal neurons and overlapping with TDP-43 pathology: positive frontal nuclear neurons (long arrow) and negative neurons (short arrow). **(E)** This 14y old C125 had frontal p-Tau plaques (arrows, brown product), same child as Da. **(F)** Third cranial nerve nucleus TDP-43 positive nuclear staining (short arrow) contrast with the lack of nuclear staining in adjacent neurons (long arrow). **(G)** This 3y old CO16 displays coiled tangles in oligodendroglia cells stained with TDP-43 (short arrow, brown product), please notice the nuclear depletion of TDP-43 immunostaining. **(H)** Same child as **(A)**, olfactory bulb glomerular region stained with alpha synuclein, red positive staining (arrows). **(I)** Substantia nigra Lewy bodies C145 in a 31y old. **(J)** Frontal cortex C170 with numerous abnormal white matter dendrites and axons positive for abnormal intermediate neurofilaments. **(K1,K2)** Olfactory nucleus in the olfactory bulb and the dentate gyrus in the hippocampus showing strongly stained p-Tau nuclei versus negative nuclei, a common finding in MMC children. **(L)** Olfactory bulb p-Tau plaque (arrows, brown product).

Brain tissues were previously investigated for the neuropathological hallmarks of AD, PD, and TDP-43 by Board certified neuropathologists and anatomical pathologists (Calderón-Garcidueñas et al., 2022a; Calderón-Garcidueñas et al., 2017; Calderón-Garcidueñas and Ayala, 2022; Calderón-Garcidueñas et al., 2024a; Calderón-Garcidueñas et al., 2024b; Calderón-Garcidueñas et al., 2018a; Calderón-Garcidueñas et al., 2020; Calderón-Garcidueñas et al., 2022c). Early subcortical and cortical lesions, hyperphosphorylated tau, amyloid-β protein phases, alpha-synuclein and TDP-43 pathology were documented (Supplementary Table S2) (Braak et al., 2011; Braak and Del Tredeci, 2015; Alafuzoff et al., 2008; Thal et al., 2002; Rahimi and Kovacs, 2014; Rüb et al., 2016; Del Tredeci and Braak, 2022; Braak et al., 2003; Jellinger, 2022). The spectrum of Alzheimer and Parkinson’s diseases along TDP-43 pathology is described in Supplementary Table S2. Our youngest 11m old baby showed already the quadruple aberrant neural pathology. Figure 16 illustrates the AD, PD, FTLD and ALS pathology in MMC children and young adults.

## 7 Discussion

AD hallmarks have been documented in 202/203 forensic autopsies in Metropolitan Mexico City residents ages 11 months to 40 years (Calderón-Garcidueñas et al., 2018a) and in a recent work, in 57 MMC children ages 14.8 ± 5.2 years, 100% had AD with an overlap of AD+TDP-43 in 43.9%, AD + PD + TDP-43 in 19.3% and AD+PD in 3.56% (Calderón-Garcidueñas et al., 2024a; Calderón-Garcidueñas et al., 2024b).

MMC highly exposed children and teens are developing Alzheimer and Parkinson’s diseases, frontal-temporal lobar degeneration and amyotrophic lateral sclerosis -all fatal diseases-, posing a public health unattended challenge. MMC neuropathology data are key for the ongoing increase in prevalence and incidence of fatal aberrant proteinopathies, as the global population grows older and complex environmental and genetic associations are at play (Dhana et al., 2023; Dauer et al., 2023; Ibanez et al., 2021; Wu et al., 2024; Mehta et al., 2023; Young et al., 2018; Kakara et al., 2024; Hendriks et al., 2024; Frisoni et al., 2024; Krzyzanowski et al., 2023; Zhang et al., 2023; Park et al., 2024; Chao, 2024; Jones et al., 2024; Yang W. et al., 2024; Ou et al., 2024; Yang Y. C. et al., 2024; Calderón-Garcidueñas et al., 2024a; Takeda, 2018; Saltiel et al., 2024; Forrest et al., 2022).

The association between magnetic, single-domain UFPM/NPs with motion behavior and the presence of quadruple neuropathological hallmarks in young urbanites is very relevant to early prevention of fatal neurodegenerative diseases and the execution of effective strategies for decreasing harmful environmental exposures (Calderón-Garcidueñas et al., 2022a; Calderón-Garcidueñas et al., 2017; Calderón-Garcidueñas and Ayala, 2022; Aguilar-Castillo, 2023; Calderón-Garcidueñas et al., 2004; Calderón-Garcidueñas et al., 2024a; Calderón-Garcidueñas et al., 2024b; Calderón-Garcidueñas et al., 2018a; Calderón-Garcidueñas et al., 2020; Calderón-Garcidueñas et al., 2022c; Calderón-Garcidueñas et al., 2022d; Takeda, 2018).

It is important to emphasize MMC residents have been exposed to PM_2.5_ ≥ 9μg/m^3^ in the last three decades, and that magnetic NPs are ubiquitous both in the city dust and in the air (Velasco et al., 2019; Caudillo et al., 2020; Dunn et al., 2004; Bautista-Hernández et al., 2023; Aguilera et al., 2021; Bautista et al., 2014; Delgado et al., 2019; Cejudo et al., 2022). Moreover, brain magnetic UFPM/NPs are documented *in utero* at PCW 12–15 (Calderón-Garcidueñas et al., 2022b).

Brain light and electron microscopy findings in young urbanites need to be analyzed in view of the neuropathology efforts to evaluate multiple brain pathologies in elderly individuals (Karanth et al., 2020; Rahimi and Kovacs, 2014; Jellinger, 2022; Takeda, 2018; Saltiel et al., 2024; Forrest et al., 2022), the extensive work in experimental magnetic field effects, and the use of magnetic NPs nanoprobes for monitoring, diagnosing and delivering targeted drugs (Kara and Ozpolat, 2024; Zhang et al., 2024; Rezaei et al., 2024; Ayansiji et al., 2020; Park et al., 2023; Soliman et al., 2024; Ruzycka-Ayoush et al., 2024; Han, 2024; Baričić et al., 2024; Moya Betancourt et al., 2024; Seino et al., 2024; Yuan et al., 2023; Hadadian et al., 2022; Ryu et al., 2022; Santoyo et al., 2011; Hosseini, 2021; Wang et al., 2019; Karkisaval et al., 2024; Sharma et al., 2024; Gomes et al., 2023; Dan et al., 2015; Imam et al., 2015; Shin and Lee, 2024; Gárate-Vélez et al., 2020; Badman et al., 2020; Ramos Docampo et al., 2024).

Magnetic particles were present in every MMC brain cortical and subcortical sample, including amygdala, olfactory bulb, optic chiasm, vagal nerves, and hypophysis, with significant SIRM regional, individual variations. The higher SIRMs documented in caudate, thalamus and hippocampus are critical, given the MRI brain atrophy, cognitive deficits, sleep and auditory disorders, and fall risk we have been documenting in seemingly healthy MMC young residents (Calderón-Garcidueñas et al., 2022c; Calderón-Garcidueñas et al., 2023a; Calderón-Garcidueñas et al., 2023b; Calderón-Garcidueñas et al., 2022e; Calderón-Garcidueñas et al., 2019). Moreover, the overlap of AD, PD, FTLD, and ALS neuropathology in children is striking in the first two decades of life (Calderón-Garcidueñas et al., 2024a). Furthermore, it is almost identical to the findings in 365 unimpaired and cognitively impaired white individuals, age 83.8 ± 8.8y from Kentucky (Karanth et al., 2020) and recognized by neuropathologists across the world as a spectrum of mixed brain pathologies beyond vascular pathology and aging (Rahimi and Kovacs, 2014; Jellinger, 2022; Calderón-Garcidueñas et al., 2024a; Takeda, 2018; Saltiel et al., 2024; Forrest et al., 2022).

The UFPM/NPs redox-active, magnetic composition and properties have been studied extensively, particularly those of iron-oxide based NPs, documented in every MMC brain (Calderón-Garcidueñas et al., 2022a; Calderón-Garcidueñas et al., 2017; Calderón-Garcidueñas and Ayala, 2022; Calderón-Garcidueñas et al., 2022b; Calderón-Garcidueñas et al., 2021; Aguilar-Castillo, 2023; Calderón-Garcidueñas et al., 2024a; Calderón-Garcidueñas et al., 2024b; Calderón-Garcidueñas et al., 2018a; Calderón-Garcidueñas et al., 2020; Calderón-Garcidueñas et al., 2022d). The cytotoxic effects of Fe NPs are very well known to cancer researchers, with and without the use of alternating magnetic fields (AMF) resulting in decreased cell viability and proliferation of cancer cells associated with magnetic fluid hyperthermia (Ruzycka-Ayoush et al., 2024). Endothelial cells (ECs) are vulnerable to magnetic NPs, causing decreased viability in primary corneal ECs alongside production of reactive oxygen species (ROS), lactate dehydrogenase, and markers of apoptosis (Park et al., 2023). The effects of magnetic UFPM/NPs include endothelial damage at the blood–brain-barrier (BBB), uncontrolled production of ROS, magnetic hyperthermia and reduced lysosomal performance (Sharma et al., 2024; Gomes et al., 2023; Dan et al., 2015; Imam et al., 2015; Shin and Lee, 2024; Gárate-Vélez et al., 2020; Badman et al., 2020). Magnetic hybrid NPs could induce temperature increases by 5°C- the minimal temperature rise required for being effectively used in hyperthermia treatments (and a serious unwanted response in a brain in development) (Gomes et al., 2023). Endothelial dysfunction is the expected outcome of oxidative vascular stress, resulting from an alteration in mitochondrial biogenesis and dynamics (Dan et al., 2015).

Brain endothelial damage is indeed an early finding in MMC children and documented in every brain region explored, with severe damage in olfactory bulb, brainstem and cerebellum (Calderón-Garcidueñas et al., 2020; Calderón-Garcidueñas et al., 2022a; Calderón-Garcidueñas et al., 2024a; Calderón-Garcidueñas et al., 2024b). Remarkably, we have documented NPs transfers from erythrocytes and/or mononuclear cells to an activated endothelium and erythrophagocytosis (i.e., Figure 14). Extensive damage to the neurovascular unit is a very early ultrafine particulate matter exposure effect.

Relevant to the early neurodegenerative effects are the anhysteretic magnetization, saturation magnetization, interparticle interactions and the fast increment of anhysteretic magnetization between 30 and 50 μT, supporting single-domain particles with distinct motion behavior. Low coercivity magnetic NPs were confirmed by IRM curves reaching saturation levels at exposures of 100–300 mT. IRM curve variation was due to particle change in position and/or orientation *in situ.*

Of utmost importance, T3 > T2 > T1 motion behavior were associated with magnetic particle’s concentrations. The highest concentrations of low coercivity magnetic minerals, i.e., high SIRM were measured in caudate, thalamus, hippocampus, putamen, and motor regions. These data are important, because all loaded magnetic NPs cells, particularly those with T2 and T3 behavior will elicit NPs motion inside critical organelles and cell structures under magnetic fields; the magnitude of the motion/deviation depending upon the magnetic field strength and intrinsic aspects of the NPs, such as membrane composition (Ramos Docampo et al., 2024). Ramos Docampo et al. (2024) demonstrated that membranes made of saturated lipids, in combination with a weak magnetic field facilitate crossing of NPs using magnetic micromotors.

Magnetophoresis must be at the core of this discussion: the motion of highly toxic, oxidative stress-producing, magnetic NPs under magnetic fields have an important effect upon effective magnetic hyperthermia, diffusion, convection, residual magnetization, and electromagnetic drift, as described by Ryu et al. (2022) and Ayansiji et al. (2020). We argue, the neural effects will depend on complex interactions between magnetic diffusion coefficients, magnetic velocity, and activity coefficients, which in turn depend on relative magnetic energy and the molar magnetic susceptibility of NPs and their composition, as extensively discussed by Ayansiji et al. (2020). Targeted highly magnetic regions in young urbanites, such as the caudate, thalamus, hippocampus, etc., are worrisome findings (Calderón-Garcidueñas et al., 2022c; Calderón-Garcidueñas and Ayala, 2022; Calderón-Garcidueñas et al., 2023a; Calderón-Garcidueñas et al., 2023b; Calderón-Garcidueñas et al., 2022e; Calderón-Garcidueñas et al., 2019).

Equally uncertain and unpredictable, are the magnetic anisotropy, saturation magnetization, and particle interactions (Baričić et al., 2024) in the different brain regions and since each neural cell is different and protein coronas-present on the surface of NPs- are also distinct in each microenvironment, *the biological identity of NPs that may be acquired after exposure to a biological matrix is mostly provided by the components of the hard corona as the pristine surface is generally less accessible for binding* (Soliman et al., 2024), thus, the ultimate NPs cytotoxicity given by the protein coronas is difficult to predict.

What is clear is that magnetic NPs enter the brain from early brain stages *in utero* (Calderón-Garcidueñas et al., 2022b) and certainly under experimental conditions—i.e., migration of PEGylated magnetic NPs getting into the brain through the nasal pathway has been confirmed by magnetization measurements (Seino et al., 2024)-, thus, portal of entry is critical in young urbanites. Hence, the observation of a significant statistical difference in subcortical versus cortical magnetic particles in MMC ≤ 40years, strongly suggests portals of entry such as the olfactory neuro-epithelium/trigeminal nerve/enteric pathways, are age-relevant, i.e., younger subjects are likely to inhale/eat/swallow UFPM/NPs that are reaching subcortical brain regions (Silver and Finger, 2009; Huff et al., 2024). We indeed have documented significant concentrations of metal-containing UFPM/NPs in trigeminal ganglia and enteric nerves of MMC children (Calderón-Garcidueñas et al., 2018b; Calderón-Garcidueñas et al., 2015).

Another critical factor for MMC residents is the association between lipopolysaccharides (LPS) and particles (Osornio-Vargas et al., 2003) and the differential exposure according to the MMC region, South > North (Osornio-Vargas et al., 2003), and MMC daily production of >13,149 tons of fecal material (an optimal LPS source) (Estrada-Garcia et al., 2002).

LPS-NPs penetrate membranes in subjects exposed to air pollutants. MMC residents are chronically exposed to LPS-NPs and either inhaling or swallowing them, they have a direct brain access and extensive inflammatory effects (Fitzgerald et al., 2004; Chen et al., 2022). Strikingly the passage of NPs is facilitated by polysaccharide-coated magnetic nanoparticles (MNPs) (Moya Betancourt et al., 2024). Moya Betancourt et al. (2024) assessed the maximum insertion pressure (MIP) and synergy of magnetite (Fe_3_O_4_) MNPs functionalized with polysaccharides into a membrane model. Key to our studies, the primary driving force of the coated MNPs incorporation into the monolayer predominantly stemmed from electrostatic interaction and the presence of a magnetic field inducing an enhancement of the insertion process of the MNPs.

This information is very pertinent to epidemiological ALS studies: environmental exposures are associated with increased ALS risk (Andrew et al., 2017; Facciponte et al., 2018; Andrew et al., 2021; Metcalf et al., 2021). Head trauma, severe electrical burns, lead hobbies, and occupational settings such as mechanics, painting, or construction have been associated with ALS (Andrew et al., 2017). Interestingly, Cyanobacteria considered Gram negative, has also been associated with ALS risk (Facciponte et al., 2018; Andrew et al., 2021; Metcalf et al., 2021). Cyanotoxins have been classified into three main classes, neurotoxins, hepatotoxins and dermatotoxins, mainly identified as peptides, alkaloids and lipopolysaccharides (Ricciardelli et al., 2023). Cyanobacteria bloom lipopolysaccharides having significant pro-inflammatory effects on epithelial and immune cells *in vitro* (Skočková et al., 2024). Based on Moya et al., (Moya Betancourt et al., 2024) findings showing the passage of NPs through membranes is facilitated by polysaccharide-coated magnetic NPs, LPS associated with Cyanobacteria and magnetic NPs could be a prime transporter into motor neurons.

Of keen interest in this work, was the significant magnetic particle concentration difference between MMC Brodmann 4 and medulla versus ALS cases with higher SIRM, along the loss of nuclear TDP-43 expression (i.e., nuclear clearing) in cortical and subcortical motor and sensory neurons in young MMC urbanites (Calderón-Garcidueñas et al., 2024b; Calderón-Garcidueñas et al., 2020; Calderón-Garcidueñas et al., 2022d). Our current MMC results suggest accumulation of motor neuron magnetic materials is an early event, single-domain Fe-NPs with high saturation magnetization likely contribute to cell toxicity and the TDP-43 alterations. A key difference between the ALS vs. Controls could be indeed the magnetic NPs profile (i.e., two intermetallic compounds, TiFe_2_ alloys) (Narayanaswamy et al., 2022; Mohapatra et al., 2013), while the nature of the nerve structure versus medullary or cortical examples could also be at play. The magnetic behavior of NPs depends on the coupling of the magnetic domains within the crystalline structure. As the dimensions decrease to nanometric sizes, the material does not support multiple domains within its structure, presenting a single domain particle, which is magnetized uniformly along its anisotropic axis, the composition and shape of the particles will impact changes in magnetization (Ma et al., 2023).

To complicate matters, action potentials in the motor neuron axon initial segment create magnetic fields, that potentially affect excitability in neurons with a load of single-domain magnetic particles with motion behaviors (Harley et al., 2023; Beros et al., 2024; Trajano et al., 2023; Schmid et al., 2024). Indeed, dysregulated neuronal excitability is the core of ALS (Harley et al., 2023). Magnetic fields could interfere with ion channel conductance, destabilizing cellular ionic homeostasis and altering h-current activity and cellular junction dynamics (Karkisaval et al., 2024; Beros et al., 2024; Trajano et al., 2023; Schmid et al., 2024; Zemel et al., 2023; Asghari et al., 2024; Wyszkowska et al., 2016). Extremely low frequency (ELF) electromagnetic fields are also likely to impact motor neuron performance. Wyszkowska et al. (2016) showed that exposure to ELF above 4 mT from an identified motor neuron- the fast extensor tibiae motor neuron in locusts-, increased spike latency, along reducing hind leg kick force, and increasing stress-protein levels (Hsp70).

Remarkably, the identification of an electromagnetic perceptive gene (EPG) in mammalian neuronal cultures, (Krishnan et al., 2018) opens the possibility of remote activation by EMF, increasing intracellular Ca concentrations and cellular excitability, certainly affecting motor neurons (Hwang et al., 2020).

The magnetic UFPM/NPs unwillingly carried by young urbanites are a fatal cargo, optimal to cause havoc and neurodegenerative progressive changes (Calderón-Garcidueñas et al., 2022a; Calderón-Garcidueñas et al., 2017; Calderón-Garcidueñas and Ayala, 2022; Calderón-Garcidueñas et al., 2022b; Calderón-Garcidueñas et al., 2021; Aguilar-Castillo, 2023; Calderón-Garcidueñas et al., 2024b; Calderón-Garcidueñas et al., 2018a; Calderón-Garcidueñas et al., 2020; Calderón-Garcidueñas et al., 2022d; Calderón-Garcidueñas et al., 2024a; Calderón-Garcidueñas et al., 2018b; Calderón-Garcidueñas et al., 2015). The work in progress of the researchers optimizing Fe oxide nanocomposites for effective hyperthermia under alternating magnetic fields (Ryu et al., 2022) is already in place in MMC brains.

Adding to our concerns, are the rotation effects of Earth-strength magnetic fields (Wang et al., 2019) upon the loaded magnetic NP neurons in pediatric populations. Wang et al. (2019) reported *termed alpha-event-related desynchronization (alpha-ERD), in response to the geomagnetic field, triggered only by horizontal rotations when the static vertical magnetic field was directed downwards, as it is in the Northern Hemisphere.*

Electromagnetic fields interact with fine and coarse black carbon particles and ducts caused by thermal inversions, resulting in super-refractive regions in the troposphere (López-Alvarez et al., 2023; Smirnov, 2023) and coal fly ash and HULIS aerosols overcoming Earth’s atmospheric flywheel (radiation buffering mechanism), along with changes in the particle flux impinging the Earth’s magnetic field are all potentially harmful for every urbanite around the globe (Whiteside and Herndon, 2024; Herndon, 2024). Magnetophoretic materials’ accumulating in tissues and their slow degradation in lysosomal environments (Torresan et al., 2021), alterations in global atmospheric electrical circuit, Schumann resonances (SRs) and the geomagnetic field are likely to impact human circadian rhythm (Martel et al., 2023). Cyclic solar disturbances, i.e., sunspots, seasonal geomagnetic field weakening and ubiquitous electromagnetic pollution (i.e., wireless devices, base antennas and low orbit internet satellites), could affect sensing of the earth’s EMFs by the human body according with Martel et al. (2023). Interestingly, space weather, i.e., solar storms, impact the Earth by disturbing the geomagnetic field. In a 22y study (Gulson-Castillo et al., 2023) of nocturnally migrating North American birds, 9 to 17% decrease in migration intensity in both spring and fall were the result of obscured celestial cues and magnetic disturbances disrupting navigation.

In the current world, we are surrounded by electromagnetic sources, for some of which we have no clue of their health effects, i.e., the new 5G infrastructure designed to utilize millimeter wave frequencies (30–300 GHz range) at data transmission rates in the order of gigabits per second (Gbps) (Redmayne and Maisch, 2023).

The American National Standards Institute (ANSI) (1998) publishes consensus standards on radio frequency (RF) exposures and measurements: *C95.6, Safety Levels with Respect to Human Exposure to Electromagnetic Fields, 0–3 kHz. Defines exposure levels to protect against adverse effects in humans from exposure to electric and magnetic fields at frequencies from 0 to 3 kHz.*

While the International Commission on Non-Ionizing Radiation Protection (ICNIRP) Guidelines [International Commission on Non-Ionizing Radiation Protection (ICNIRP) Guidelines, 1998] establishes guidelines for the protection of workers moving in static magnetic fields or being exposed to magnetic fields with frequencies below 1 Hz. The ICNIRP is an affiliate of the World Health Organization (WHO). Interestingly, the 2005 WHO Task Group of scientific experts to assess any risks to health that might exist from exposure to ELF electric and magnetic fields in the frequency range > 0 to 100,000 Hz (100 kHz) [World Health Organization (WHO), n.d.] concluded that there *are no substantive health issues related to ELF electric fields at levels generally encountered by members of the public*. WHO agreed all populations are now exposed to varying degrees of EMF, and the levels will continue to increase as technology advances [World Health Organization (WHO), n.d.].

For the purposes of the EMF Project, the exposure range is divided as static (0 Hz), extremely low frequency (ELF, >0–300 kHz), intermediate frequencies (IF, >300 Hz to 10 MHz), and radiofrequency (RF, 10 MHz–300 GHz) fields. Unfortunately, in a recent meta-analysis paper, partially funded by the WHO radioprotection program, their conclusions were *inherent limitations of the research results in substantial uncertainty* (Röösli et al., 2024).

The ultimate importance of iron NPs composed of magnetite and maghemite was discussed by Yarjanli et al. (2017): *their hydrodynamic radius and surface charge regulate their time in circulation and accessibility to tissues and cell up-take*. Equally critical is the degree of NPs’ crystallinity and magnetic responses as superbly described by Pacakova et al. (2017).

Gutiérrez et al. (2019) work is very important in this discussion: iron oxide NPs aggregation and agglomeration impact their magnetic and heating properties. Vital to their capacity to cause cell damage is their single-domain and their size. Her group used 14 and 22 nm magnetic NPs with the same core but subjected to different surface modifications procedures, their results were NPs with different size aggregates and arrangements.

Gutiérrez et al. (2019) also discussed the NPs factors accounting for cytotoxicity: (a) Colloidal stability depending on the balance of magnetic, dipolar and van der Waals forces and repulsive interactions, mainly electrostatic and steric. (b) The assembly of many magnetic cores, the distance between cores affecting the magnetic behavior of the entire particle. (c) Dipole–dipole interactions –depending on inter-particle distances-result in an alteration of magnetic properties. High concentrations of magnetic NPs in organelles such as endosomes, mitochondria, endoplasmic reticulum, etc., elicits strong magnetic interactions and exposures to AMF give rise to heat. (d) The protein corona-NP binding affinity is also key, shape, size and surface characteristics of NPs and forces such as hydrodynamic, electrodynamic and magnetic, all determine their capacity to enter cells, trafficking, and biodistribution (Kim et al., 2016).

Strikingly, it is well known NPs can perform as molecular chaperons to conduct protein folding, destabilization and protein aggregates (Parveen et al., 2017). This is a critical issue when the hydrophobic protein residues get exposed to an aqueous environment. The formation of fibrils is a nucleation-dependent process, and you need a critical nucleus that is followed by enhanced rate of protein fibrillation (Linse et al., 2007). Lipid bilayers, collagen fibers, and polysaccharides covered surfaces are very effective in promoting amyloid formation. Protein monomers participate in the seed-independent homogeneous nucleation- and-growth mechanism, favored by low pH <3 (Linse et al., 2007). NPs, crystals, and fibrils catalyze the generation of new aggregates on their surface and this secondary nucleation process can be many orders of magnitude faster than primary nucleation (Thacker et al., 2020).

In AD, secondary nucleation leads to multiplication and propagation of aggregates, thus short-lived oligomeric intermediates cause neurotoxicity (Thacker et al., 2020). The propagation of amyloid fibril strains is possible in systems dominated by secondary nucleation rather than fragmentation (Thacker et al., 2020). Either acceleration or inhibition of fibril amyloid formation is accomplished by a change in NPs size and curvature (John et al., 2022). Differences in peptide NP effects resulted from different peptide properties (size, tendency to aggregate) and associated surface binding affinities (John et al., 2022). And very important to understand the complexity of NPs neural effects: high surface NPs curvatures destabilized prefibrillar structures, a potential explanation for inhibitory effects on fibril growth, provided that peptide-NP surface binding was relevant for fibril formation (John et al., 2022). NPs capable of accelerating protein oligomerization should be considered neurotoxic and neurodegenerative, i.e., fabricated CuO NPs ~ 50 nm led to an acceleration in Aβ_1-42_ oligomerization in a concentration-dependent manner through shortening the nucleation step and promoting the fibrillization rate (Jaragh-Alhadad and Falahati, 2022).

Keep in mind, amyloid-prone proteins are small and *intrinsically disordered in non-aggregated states* (Hartl, 2017). Oligomers expose sticky surfaces -very efficient in disturbing phospholipid layers- and interact with cellular proteins (Chiti and Dobson, 2017; Olzscha et al., 2011). Olzscha et al. (2011) discussed *the capacity of the amyloid-like aggregates to promote aberrant protein interactions and to deregulate the cytosolic stress response.* The targets are proteins involved in chromatin organization, transcription, translation, maintenance of cell architecture and protein quality control (Olzscha et al., 2011), a decidedly relevant finding when we see their association with neurodegeneration in young individuals (Calderón-Garcidueñas et al., 2018a; Calderón-Garcidueñas et al., 2020; Calderón-Garcidueñas et al., 2022d; Calderón-Garcidueñas et al., 2024a). Sontag et al. (2017) described the key role of proteostasis pathways as a network of molecular chaperons, and critical clearance pathways involved in the recognition, refolding and clearance of aberrant proteins.

Protein quality control (PQC) is of extreme importance in neurodegeneration (Sontag et al., 2017; Neal et al., 2022; Escusa-Toret et al., 2013; Rolli and Sontag, 2022; Gerson et al., 2022; Johnston and Samant, 2021; Torkzaban et al., 2020) anything interfering with PQC will result in cell damage and likewise damaged organelles in charge of handling misfolding and stress-damaged proteins in route to clearance by the ubiquitin-proteasome system (UPS) i.e., endoplasmic reticulum, will have the same harmful effect (Escusa-Toret et al., 2013).

TiO2-NPs for example, enhance α synuclein fibril formation and SiO2-NPs (both very common NPs in MMC brains) boost α-synuclein aggregation kinetics in a dose-dependent manner and induced oxidative stress and α-synuclein aggregation by inhibiting the ubiquitin-proteasome system UPS in PC12 cells, a dopaminergic neuron-like cell line (Mohammadi and Nikkhah, 2017; Vitali et al., 2018; Xie and Wu, 2016). Particle size and surface coating charge impacted amyloid β fibrillation in a model using dextran-coated superparamagnetic iron oxide NPs (Mahmoudi et al., 2013). Positively charged NPs can promote fibrillation at significantly lower NPs concentrations (Mahmoudi et al., 2013).

Interestingly, trans-neuronal tau spreading does not depend on tau’s aggregation propensity/misfolding and does not lead to templated misfolding in recipient neurons (Rodrigues et al., 2023), thus we strongly support NPs transsynaptic transport will be the most likely pathway for aberrant protein formation. Recent literature (Karaagac and Köçkar, 2012; Köçkar et al., 2019; Karaagac and Köçkar, 2022) addressing the interaction of FeNPs in the air, the effects of surfactants upon magnetic NPs properties, and the impact on saturation magnetization using coated superparamagnetic iron oxide NPs with polyethylene glycol (PEG)- a commonly used pharmaceutical excipient in oral, topical and parenteral products-, will help neuronanotoxicologists to understand the complexity of the harmful effects of magnetic NPs in the human brain.

## 8 Summary

Magnetic UFPM/NPs likely play a key role in the early development and progression of AD, PD, FTLD and ALS in young urbanites exposed to ubiquitous anthropogenic air pollution, ultrafine and industrial magnetic particles, and electromagnetic fields.

The neurovascular unit is an early target of UFPM/NPs and magnetic particle concentrations and motion behavior explain TEM subcellular pathology and brain MRI structural and volumetric alterations in young urbanites, along with the significant cognitive, olfaction, auditory and gait and equilibrium abnormalities.

Fe NPs and their toxic alloys are ubiquitous in urban young brains. Young urbanites have significant accumulation of magnetic subcortical NPs, an issue of major concern because they inhale, and swallow UFPM/NPs and they are massively exposed to magnetic fields.

There are key questions that we need to address: (1) What are the primary driving forces for magnetophoresis in the central nervous system? (2) How do particle interactions, size, shape and chemical composition affect magnetic neural targeting? (3) What is the impact of magnetic fields upon the cytotoxic effects, including hyperthermia and particle motion? (4) What NPs properties, apart from composition and shape, are key to cause the most CNS damage? (5) What neural subcellular characteristics make targeted neurons more vulnerable to magnetophoresis? (6) Hyperphosphorylated tau -is invariable present in all children and young adults in MMC- we question: is this key aberrant protein particularly susceptible to magnetic effects? (7) Quadruple aberrant pathology seen in pediatric ages is very close to the one seen 70y later, so what determines some patients get AD and others PD, ALS or/and FTLD? (8) What are the genetic factors affecting the severity and/or propagation of neural damage due to magnetic nanoscale particulates, (9) Is there a threshold for exposures to electromagnetic fields? (10) Exposures to magnetic NPs and electromagnetic fields are ubiquitous, they are getting worse, so how are going to protect our populations?

It is urgent to implement a monitoring system for NPs in the air, soil, plants, and urban dust. Magnetic UFPM/NPs are a fatal brain cargo in pediatric ages and an environmental threat. Billions of people are at risk. We are clearly poisoning ourselves.

## Data Availability

The original contributions presented in the study are included in the article/Supplementary material, further inquiries can be directed to the corresponding author.
