# Supplementary material for: Single-domain magnetic particles with motion behavior under electromagnetic AC and DC fields are a fatal cargo in Metropolitan Mexico City pediatric and young adult early Alzheimer, Parkinson, frontotemporal lobar degeneration and amyotrophic lateral sclerosis and in ALS patients
Source: Front Hum Neurosci. 2024 Aug 23;18:1411849. doi: 10.3389/fnhum.2024.1411849 (PMC11377271; doi:10.3389/fnhum.2024.1411849)
Supplement: Supplementary file 1 [file Table_1.DOCX]

Supplementary Material

# Supplementary Table 1

Table 1 Supplemental. Brain samples with individuals age, sex, anatomical sites and magnetic variables.

| sample ID | AGE | SEX | ANAT SITE | weight | ρ | ARM_50_ | S-_300_ | IRM__1000_ | IRM__-300_ |
| --- | --- | --- | --- | --- | --- | --- | --- | --- | --- |
|  |  |  |  | g | kg/m3 | μAm2/kg | Adim | μAm2/kg | μAm2/kg |
| C001 | 25 | 0 | TEMPORAL | 1.1 | 140.0 | 0.13 | 0.99 | 9.48 | 9.37 |
| C002 | 55 | 0 | TEMPORAL | 1.4 | 171.3 | 0.11 | 1.00 | 8.77 | 9.04 |
| C003 | 55 | 1 | TEMPORAL | 0.4 | 56.2 | 0.20 | 0.85 | 16.85 | 14.28 |
| C004 | 55 | 1 | TEMPORAL | 0.5 | 63.8 | 0.67 | 0.96 | 35.80 | 34.39 |
| C005 | 90 | 0 | TEMPORAL | 1.3 | 165.0 | 0.68 | 1.00 | 70.36 | 72.65 |
| C006 | 41 | 1 | TEMPORAL | 1.1 | 140.0 | 0.13 | 0.98 | 12.80 | 12.55 |
| C007 | 29 | 1 | TEMPORAL | 0.8 | 97.5 | 0.16 | 0.98 | 15.54 | 15.17 |
| C008 | 39 | 1 | TEMPORAL | 1.2 | 147.5 | 0.24 | 0.99 | 19.47 | 19.19 |
| C009 | 53 | 1 | TEMPORAL | 0.9 | 118.8 | 0.15 | 1.00 | 10.76 | 10.77 |
| C010 | 27 | 1 | TEMPORAL | 0.9 | 113.8 | 0.13 | 1.00 | 11.66 | 11.71 |
| C011 | 65 | 0 | TEMPORAL | 0.6 | 76.2 | 0.25 | 0.95 | 14.09 | 13.38 |
| C012 | 3 | 1 | MEDULLA | 0.2 | 28.7 | 0.37 | 0.97 | 33.81 | 32.70 |
| C013 | 3 | 1 | PUTAMEN | 0.5 | 62.5 | 0.30 | 0.96 | 34.60 | 33.32 |
| C014 | 3 | 1 | FRONTAL | 0.3 | 38.8 | 0.34 | 0.85 | 27.02 | 23.07 |
| C015 | 3 | 1 | PONS UPPER | 1.0 | 126.3 | 0.25 | 1.00 | 26.57 | 26.48 |
| C016 | 3 | 1 | TEMPORAL | 0.6 | 76.2 | 0.19 | 0.98 | 14.85 | 14.52 |
| C017 | 3 | 1 | CEREBELLUM | 0.7 | 87.5 | 0.29 | 1.00 | 21.62 | 21.69 |
| C018 | 3 | 1 | CAUDATE | 0.2 | 30.0 | 0.64 | 0.98 | 55.19 | 53.82 |
| C019 | 3 | 1 | MIDBRAIN | 0.2 | 20.0 | 1.80 | 1.00 | 135.09 | 135.09 |
| C020 | 1 | 1 | CEREBELLUM | 0.7 | 83.8 | 0.27 | 1.00 | 18.90 | 18.99 |
| C021 | 1 | 1 | CAUDATE | 0.4 | 52.5 | 0.42 | 0.98 | 24.27 | 23.81 |
| C022 | 1 | 1 | HIPPOCAMPUS | 0.2 | 21.3 | 0.65 | 0.63 | 56.46 | 35.46 |
| C023 | 1 | 1 | CERVICAL cord | 0.1 | 12.5 | 1.55 | 0.97 | 122.09 | 118.36 |
| C024 | 1 | 1 | OCCIPITAL | 0.1 | 15.0 | 0.73 | 0.98 | 44.09 | 43.05 |
| C025 | 1 | 1 | PONS UPPER | 0.3 | 40.0 | 0.54 | 1.00 | 32.06 | 32.13 |
| C026 | 1 | 1 | FRONTAL | 0.1 | 13.7 | 1.12 | 0.97 | 63.65 | 61.65 |
| C027 | 1 | 1 | TEMPORAL | 0.5 | 62.5 | 0.23 | 1.00 | 18.36 | 19.10 |
| C028 | 24 | 1 | FRONTAL | 0.8 | 105.0 | 0.17 | 0.99 | 14.39 | 14.18 |
| C029 | 24 | 1 | CEREBELLUM | 0.7 | 86.2 | 0.16 | 0.97 | 10.26 | 10.00 |
| C030 | 24 | 1 | CAUDATE | 0.7 | 86.2 | 2.51 | 1.00 | 73.93 | 74.00 |
| C031 | 24 | 1 | MIDBRAIN | 0.4 | 55.0 | 0.36 | 1.00 | 27.80 | 32.77 |
| C032 | 24 | 1 | PUTAMEN | 0.5 | 65.0 | 0.16 | 0.87 | 16.39 | 14.26 |
| C033 | 24 | 1 | BRODMANN 4 | 0.5 | 63.8 | 0.36 | 0.96 | 42.08 | 40.48 |
| C034 | 24 | 1 | TEMPORAL | 0.6 | 77.5 | 0.25 | 0.97 | 20.38 | 19.83 |
| C035 | 24 | 1 | PONS | 0.5 | 66.2 | 0.32 | 0.97 | 30.40 | 29.64 |
| C036 | 13 | 0 | TEMPORAL | 0.5 | 61.2 | 0.26 | 1.00 | 19.19 | 19.33 |
| C037 | 13 | 0 | FRONTAL | 1.0 | 120.0 | 0.11 | 1.00 | 6.81 | 6.84 |
| C038 | 13 | 0 | HIPPOCAMPUS | 0.6 | 81.2 | 0.17 | 0.98 | 12.36 | 12.15 |
| C039 | 13 | 0 | CAUDATE | 0.6 | 78.8 | 0.14 | 0.98 | 10.92 | 10.75 |
| C040 | 13 | 0 | CEREBELLUM | 0.4 | 55.0 | 0.26 | 0.97 | 15.32 | 14.85 |
| C041 | 16 | 1 | HIPPOCAMPUS | 0.3 | 38.8 | 0.58 | 0.98 | 74.98 | 73.59 |
| C042 | 16 | 1 | CEREBELLUM | 0.5 | 60.0 | 0.22 | 0.96 | 18.39 | 17.71 |
| C043 | 16 | 1 | THALAMUS | 0.3 | 35.0 | 4.99 | 1.00 | 290.93 | 516.37 |
| C044 | 16 | 1 | CAUDATE | 0.3 | 36.3 | 0.48 | 0.98 | 31.08 | 30.36 |
| C045 | 16 | 1 | TEMPORAL | 0.4 | 45.0 | 0.07 | 1.00 | 31.48 | 98.76 |
| C046 | 16 | 1 | CERVICAL cord | 0.5 | 58.8 | 0.64 | 0.98 | 29.21 | 28.48 |
| C047 | 16 | 1 | PUTAMEN | 0.2 | 28.7 | 0.68 | 1.00 | 54.17 | 54.22 |
| C048 | 16 | 1 | PONS | 0.4 | 53.8 | 0.27 | 0.99 | 18.92 | 18.72 |
| C049 | 25 | 0 | PUTAMEN | 0.7 | 90.0 | 0.19 | 0.98 | 9.16 | 8.99 |
| C050 | 25 | 0 | CEREBELLUM | 1.0 | 127.5 | 0.19 | 0.96 | 9.81 | 9.46 |
| C051 | 25 | 0 | CAUDATE | 1.0 | 120.0 | 0.10 | 0.97 | 9.11 | 8.80 |
| C052 | 25 | 0 | TEMPORAL | 1.3 | 166.3 | 0.13 | 0.97 | 7.43 | 7.19 |
| C053 | 25 | 0 | THALAMUS | 0.7 | 87.5 | 0.14 | 0.98 | 11.31 | 11.06 |
| C054 | 25 | 0 | PONS | 0.8 | 98.8 | 0.16 | 0.98 | 11.91 | 11.64 |
| C055 | 25 | 0 | HIPPOCAMPUS | 1.2 | 150.0 | 0.08 | 1.00 | 8.96 | 8.94 |
| C056 | 25 | 0 | MEDULLA | 0.4 | 47.5 | 0.28 | 0.96 | 19.86 | 19.13 |
| C057 | 70 | 1 | BRODMANN 4 | 0.3 | 36.3 | 0.41 | 0.96 | 26.84 | 25.88 |
| C058 | 70 | 1 | MEDULLA | 0.2 | 25.0 | 1.12 | 1.00 | 85.26 | 102.08 |
| C059 | 70 | 1 | BRODMANN 4 | 0.3 | 32.5 | 0.68 | 0.73 | 46.46 | 33.78 |
| C060 | 71 | 1 | MEDULLA | 0.1 | 13.7 | 0.83 | 0.98 | 69.40 | 68.15 |
| C061 | 71 | 1 | BRODMANN 4 | 0.2 | 22.5 | 0.70 | 0.84 | 51.52 | 43.40 |
| C062 | 73 | 1 | MEDULLA | 0.2 | 20.0 | 0.78 | 1.00 | 35.70 | 48.40 |
| C063 | 73 | 1 | BRODMANN 4 | 0.2 | 23.7 | 0.38 | 0.98 | 33.18 | 32.57 |
| C064 | 74 | 1 | BRODMANN 4 | 0.1 | 10.0 | 2.34 | 1.00 | 77.24 | 135.77 |
| C065 | 68 | 1 | MEDULLA L | 0.1 | 18.7 | 6.67 | 0.25 | 330.53 | 83.46 |
| C066 | 74 | 1 | MEDULLA | 0.1 | 7.5 | 1.94 | 0.98 | 116.26 | 113.65 |
| C067 | 74 | 1 | MEDULLA | 0.2 | 20.0 | 0.71 | 0.66 | 62.71 | 41.67 |
| C068 | 74 | 1 | BRODMANN 4 CTL | 0.2 | 25.0 | 0.61 | 0.98 | 41.25 | 40.37 |
| C069 | 73 | 1 | BRODMANN 4 ALS | 0.5 | 63.8 | 0.30 | 0.99 | 17.66 | 17.41 |
| C070 | 74 | 1 | BRODMANN 4 CTL | 0.5 | 60.0 | 0.25 | 1.00 | 17.28 | 20.73 |
| C071 | 71 | 1 | BRODMANN 4 ALS | 0.3 | 33.7 | 0.47 | 0.99 | 29.38 | 29.18 |
| C072 | 72 | 1 | BRODMANN 4 CTL | 0.4 | 51.2 | 0.13 | 1.00 | 17.26 | 17.31 |
| C073 | 80 | 1 | BRODMANN 4 ALS | 0.3 | 37.5 | 0.37 | 1.00 | 21.04 | 21.47 |
| C074 | 68 | 1 | BRODMANN 4 ALS | 0.2 | 23.7 | 1.11 | 1.00 | 51.86 | 51.76 |
| C075 | 79 | 1 | BRODMANN 4 ALS | 0.2 | 20.0 | 0.73 | 0.97 | 54.32 | 52.70 |
| C076 | 88 | 1 | BRODMANN 4 ALS | 0.2 | 20.0 | 0.69 | 0.99 | 60.71 | 60.32 |
| C077 | 80 | 1 | CERV CORD ALS | 0.1 | 10.0 | 1.14 | 0.99 | 75.06 | 73.97 |
| C078 | 74 | 1 | MEDULLA CTL | 0.6 | 73.8 | 0.18 | 0.99 | 11.40 | 11.28 |
| C079 | 79 | 1 | MEDULLA ALS | 0.2 | 27.5 | 1.04 | 1.00 | 57.65 | 59.03 |
| C080 | 88 | 1 | MEDULLA ALS | 0.3 | 33.7 | 0.43 | 1.00 | 25.84 | 26.34 |
| C081 | 68 | 1 | MEDULLA ALS FTLD | 0.1 | 15.0 | 0.97 | 0.64 | 75.56 | 48.07 |
| C082 | 72 | 1 | MEDULLA CTL | 0.1 | 16.3 | 0.80 | 0.90 | 39.50 | 35.46 |
| C083 | 71 | 1 | MEDULLA ALS | 0.2 | 21.3 | 0.57 | 0.99 | 61.24 | 60.63 |
| C084 | 88 | 1 | PONS ALS | 0.2 | 26.3 | 0.56 | 0.99 | 28.73 | 28.37 |
| C085 | 17 | 1 | CEREBELLUM | 1.2 | 153.8 | 0.12 | 0.95 | 14.77 | 14.09 |
| C086 | 17 | 1 | TEMPORAL | 1.3 | 163.8 | 0.07 | 0.96 | 4.87 | 4.69 |
| C087 | 17 | 1 | FRONTAL | 1.8 | 221.3 | 0.07 | 0.96 | 5.74 | 5.53 |
| C088 | 45 | 1 | ANT CINGULATE | 0.7 | 92.5 | 0.20 | 0.98 | 10.88 | 10.67 |
| C089 | 45 | 1 | TEMPORAL | 1.2 | 146.3 | 0.09 | 0.97 | 7.09 | 6.85 |
| C090 | 45 | 1 | CEREBELLUM | 0.8 | 96.2 | 0.17 | 0.97 | 9.62 | 9.30 |
| C091 | 45 | 1 | CEREBELLUM | 0.7 | 86.2 | 0.59 | 1.00 | 55.29 | 55.48 |
| C092 | 45 | 1 | PUTAMEN | 1.1 | 133.8 | 0.08 | 0.95 | 5.35 | 5.07 |
| C093 | 45 | 1 | CAUDATE | 0.6 | 78.8 | 0.15 | 0.97 | 8.29 | 8.08 |
| C094 | 54 | 1 | ANT CINGULATE | 0.8 | 93.8 | 0.09 | 0.65 | 10.30 | 6.69 |
| C095 | 54 | 1 | PUTAMEN | 1.0 | 123.8 | 0.13 | 0.95 | 8.23 | 7.81 |
| C096 | 54 | 1 | CAUDATE | 0.6 | 81.2 | 0.17 | 0.95 | 10.74 | 10.19 |
| C097 | 54 | 1 | BRODMANN 4 | 0.8 | 102.5 | 0.10 | 0.97 | 7.91 | 7.66 |
| C098 | 54 | 1 | TEMPORAL | 0.9 | 106.3 | 0.13 | 1.00 | 8.46 | 10.79 |
| C099 | 54 | 1 | CEREBELLUM | 1.1 | 131.3 | 0.13 | 0.96 | 8.15 | 7.86 |
| C100 | 17 | 1 | CALCARINE | 0.5 | 62.5 | 0.18 | 0.99 | 13.52 | 13.43 |
| C101 | 17 | 1 | CEREBELLUM | 0.7 | 92.5 | 0.09 | 1.00 | 8.48 | 8.57 |
| C102 | 17 | 1 | PUTAMEN | 0.5 | 63.8 | 0.27 | 1.00 | 15.27 | 15.37 |
| C103 | 17 | 1 | ANT CINGULATE | 1.0 | 120.0 | 0.07 | 0.97 | 6.33 | 6.15 |
| C104 | 17 | 1 | BRODMANN 4 | 0.7 | 90.0 | 0.16 | 0.98 | 12.88 | 12.58 |
| C105 | 35 | 1 | BRODMANN 4 | 0.7 | 90.0 | 0.19 | 0.94 | 24.78 | 23.23 |
| C106 | 35 | 1 | CEREBELLUM | 0.7 | 83.8 | 0.32 | 1.00 | 25.41 | 25.43 |
| C107 | 35 | 1 | CAUDATE | 0.4 | 53.8 | 0.40 | 0.99 | 54.49 | 54.20 |
| C108 | 35 | 1 | FRONTAL | 1.3 | 158.8 | 0.07 | 0.98 | 3.92 | 3.82 |
| C109 | 35 | 1 | THALAMUS | 1.3 | 163.8 | 0.08 | 1.00 | 18.27 | 18.88 |
| C110 | 35 | 1 | TEMPORAL | 1.0 | 128.8 | 0.09 | 0.98 | 6.17 | 6.04 |
| C111 | 35 | 1 | PUTAMEN | 0.8 | 93.8 | 0.11 | 0.95 | 8.32 | 7.87 |
| C112 | 35 | 1 | ANT CINGULATE | 0.4 | 47.5 | 0.20 | 0.97 | 17.03 | 16.47 |
| C113 | 17 | 1 | FRONTAL | 1.5 | 192.5 | 0.23 | 0.99 | 16.27 | 16.05 |
| C114 | 17 | 1 | CAUDATE | 0.2 | 23.7 | 0.79 | 0.97 | 83.26 | 80.97 |
| C115 | 17 | 1 | PUTAMEN | 0.9 | 108.8 | 0.11 | 0.98 | 10.86 | 10.66 |
| C116 | 17 | 1 | ANT CINGULATE | 0.6 | 72.5 | 0.24 | 0.97 | 14.75 | 14.27 |
| C117 | 17 | 1 | HIPPOCAMPUS | 0.5 | 61.2 | 0.21 | 0.98 | 19.96 | 19.61 |
| C118 | 17 | 1 | CEREBELLUM | 1.1 | 140.0 | 0.11 | 0.84 | 7.65 | 6.44 |
| C119 | 17 | 1 | THALAMUS | 1.0 | 126.3 | 0.11 | 0.97 | 7.39 | 7.16 |
| C120 | 17 | 1 | TEMPORAL | 0.7 | 82.5 | 0.16 | 0.96 | 16.15 | 15.54 |
| C121 | 14 | 1 | CEREBELLUM | 1.1 | 137.5 | 0.09 | 0.96 | 5.09 | 4.89 |
| C122 | 14 | 1 | TEMPORAL | 0.8 | 105.0 | 0.28 | 0.99 | 36.58 | 36.14 |
| C123 | 14 | 1 | BRODMANN 4 | 1.2 | 153.8 | 0.10 | 0.67 | 8.64 | 5.82 |
| C124 | 14 | 1 | THALAMUS | 0.7 | 91.2 | 0.30 | 1.00 | 16.84 | 17.25 |
| C125 | 14 | 1 | FRONTAL | 0.9 | 111.3 | 0.10 | 1.00 | 6.80 | 7.04 |
| C126 | 14 | 1 | AMYGDALA | 0.8 | 95.0 | 0.19 | 1.00 | 10.71 | 10.86 |
| C127 | 58 | 1 | CEREBELLUM | 0.8 | 96.2 | 0.12 | 0.96 | 7.21 | 6.94 |
| C128 | 58 | 1 | PUTAMEN | 0.6 | 73.8 | 0.12 | 0.96 | 9.40 | 9.04 |
| C129 | 58 | 1 | FRONTAL | 1.0 | 120.0 | 0.06 | 0.99 | 10.57 | 10.47 |
| C130 | 58 | 1 | TEMPORAL | 1.0 | 121.3 | 0.14 | 0.94 | 7.35 | 6.92 |
| C131 | 58 | 1 | CAUDATE | 0.4 | 46.3 | 0.24 | 0.97 | 19.63 | 18.99 |
| C132 | 58 | 1 | BRODMANN 4 | 0.8 | 96.2 | 0.11 | 0.97 | 7.57 | 7.33 |
| C133 | 58 | 1 | SUBST NIGRAE | 0.4 | 47.5 | 0.25 | 0.97 | 13.92 | 13.47 |
| C134 | 58 | 1 | ANT CINGULATE | 0.9 | 115.0 | 0.11 | 0.97 | 6.66 | 6.45 |
| C135 | 22 | 0 | CEREBELLUM | 0.5 | 62.5 | 0.41 | 0.92 | 18.50 | 17.05 |
| C136 | 22 | 0 | BRODMANN 4 | 0.8 | 93.8 | 0.14 | 1.00 | 9.00 | 9.32 |
| C137 | 22 | 0 | TEMPORAL | 1.1 | 132.5 | 0.44 | 0.93 | 59.02 | 54.65 |
| C138 | 22 | 0 | THALAMUS | 0.4 | 53.8 | 0.22 | 0.95 | 13.59 | 12.89 |
| C139 | 31 | 1 | PUTAMEN | 1.1 | 132.5 | 0.10 | 0.97 | 5.29 | 5.14 |
| C140 | 31 | 1 | CAUDATE | 0.2 | 23.7 | 0.53 | 0.97 | 31.27 | 30.27 |
| C141 | 31 | 1 | RED NUCLEUS | 0.6 | 81.2 | 0.17 | 0.98 | 9.74 | 9.52 |
| C142 | 31 | 1 | RED NUCLEUS | 0.9 | 107.5 | 0.08 | 0.98 | 8.39 | 8.21 |
| C143 | 31 | 1 | ANT CINGULATE | 1.0 | 127.5 | 0.07 | 0.98 | 12.30 | 12.04 |
| C144 | 31 | 1 | BRODMANN 4 | 1.0 | 123.8 | 0.12 | 1.00 | 7.41 | 7.46 |
| C145 | 31 | 1 | SUBST NIGRAE | 0.4 | 46.3 | 0.26 | 0.97 | 23.67 | 23.02 |
| C146 | 27 | 1 | CEREBELLUM | 1.1 | 136.3 | 0.10 | 0.97 | 6.79 | 6.57 |
| C147 | 27 | 1 | ANT CINGULATE | 0.4 | 43.8 | 0.24 | 0.98 | 17.56 | 17.29 |
| C148 | 27 | 1 | CAUDATE | 0.2 | 27.5 | 36.38 | 0.99 | 800.79 | 790.94 |
| C149 | 27 | 1 | PUTAMEN | 0.4 | 46.3 | 3.46 | 0.99 | 174.32 | 173.06 |
| C150 | 27 | 1 | THALAMUS | 0.3 | 32.5 | 0.52 | 0.85 | 86.89 | 74.29 |
| C151 | 20 | 1 | ANT CINGULATE | 0.8 | 101.3 | 0.12 | 0.96 | 13.35 | 12.86 |
| C152 | 20 | 1 | PUTAMEN | 0.2 | 27.5 | 0.27 | 0.98 | 36.70 | 36.14 |
| C153 | 20 | 1 | TEMPORAL | 0.6 | 77.5 | 0.18 | 1.00 | 8.69 | 8.68 |
| C154 | 20 | 1 | CAUDATE | 0.1 | 15.0 | 1.49 | 1.00 | 41.22 | 43.55 |
| C155 | 20 | 1 | CEREBELLUM | 0.5 | 65.0 | 0.18 | 1.00 | 14.00 | 14.01 |
| C156 | 63 | 1 | TEMPORAL | 0.5 | 57.5 | 0.32 | 0.94 | 10.64 | 10.00 |
| C157 | 63 | 1 | CAUDATE | 0.1 | 17.5 | 0.92 | 0.90 | 41.04 | 36.96 |
| C158 | 63 | 1 | ANT CINGULATE | 0.6 | 72.5 | 0.07 | 0.98 | 8.08 | 7.92 |
| C159 | 63 | 1 | PUTAMEN | 0.7 | 90.0 | 0.22 | 0.97 | 11.87 | 11.53 |
| C160 | 63 | 1 | BRODMANN 4 | 1.1 | 133.8 | 0.09 | 1.00 | 8.24 | 8.34 |
| C161 | 36 | 0 | AMYGDALA | 0.4 | 53.8 | 0.21 | 0.98 | 9.18 | 9.00 |
| C162 | 36 | 0 | TEMPORAL | 0.7 | 83.8 | 0.17 | 0.92 | 10.52 | 9.70 |
| C163 | 36 | 0 | PUTAMEN | 0.5 | 62.5 | 0.28 | 0.97 | 13.84 | 13.37 |
| C164 | 36 | 0 | HYPOTHALAMUS | 0.1 | 18.7 | 0.71 | 0.96 | 29.87 | 28.79 |
| C165 | 36 | 0 | ANT CINGULATE | 0.8 | 102.5 | 0.15 | 0.96 | 5.00 | 4.79 |
| C166 | 36 | 0 | BRODMANN 4 | 0.7 | 92.5 | 0.35 | 0.97 | 25.95 | 25.14 |
| C167 | 36 | 0 | THALAMUS | 0.4 | 45.0 | 0.73 | 1.00 | 16.79 | 17.13 |
| C168 | 36 | 0 | GLOBUS pallidus | 0.4 | 51.2 | 0.26 | 1.00 | 17.04 | 17.11 |
| C169 | 36 | 0 | CAUDATE | 0.1 | 10.0 | 1.02 | 0.95 | 57.66 | 55.03 |
| C170 | 36 | 0 | BRODMANN 4 | 0.4 | 45.0 | 0.24 | 1.00 | 12.72 | 12.81 |
| C171 | 36 | 0 | CEREBELLUM | 0.4 | 56.2 | 0.17 | 1.00 | 6.80 | 8.08 |
| C172 | 72 | 1 | FRONTAL | 1.0 | 122.5 | 0.08 | 1.00 | 5.15 | 5.20 |
| C173 | 72 | 1 | PUTAMEN | 0.9 | 111.3 | 0.09 | 0.99 | 13.02 | 12.94 |
| C174 | 72 | 1 | CAUDATE | 0.0 | 1.2 | 7.16 | 1.00 | 470.57 | 660.84 |
| C175 | 18 | 1 | PUTAMEN | 0.9 | 111.3 | 0.07 | 0.97 | 9.18 | 8.88 |
| C176 | 18 | 1 | CAUDATE | 0.9 | 111.3 | 0.05 | 1.00 | 4.10 | 4.19 |
| C177 | 18 | 1 | BRODMANN 4 | 1.1 | 136.3 | 0.08 | 0.98 | 3.48 | 3.41 |
| C178 | 18 | 1 | CEREBELLUM | 0.7 | 87.5 | 0.13 | 1.00 | 5.73 | 5.82 |
| C179 | 18 | 1 | GLOBUS pallidus | 1.2 | 148.8 | 0.08 | 0.98 | 5.81 | 5.67 |
| C180 | 21 | 1 | PONS UPPER | 0.5 | 63.8 | 0.14 | 0.98 | 23.86 | 23.44 |
| C181 | 21 | 1 | TECTUM | 0.3 | 41.3 | 0.08 | 0.90 | 13.98 | 12.63 |
| C182 | 21 | 1 | BRODMANN 4 | 0.5 | 67.5 | 0.37 | 0.99 | 44.49 | 44.05 |
| C183 | 21 | 1 | BRODMANN 4 | 0.9 | 110.0 | 0.07 | 0.99 | 5.41 | 5.35 |
| C184 | 21 | 1 | CAUDATE | 0.2 | 23.7 | 0.64 | 1.00 | 27.17 | 27.85 |
| C185 | 21 | 1 | ANT CINGULATE | 1.1 | 140.0 | 0.05 | 0.97 | 5.60 | 5.44 |
| C186 | 21 | 1 | CEREBELLUM | 0.8 | 96.2 | 0.48 | 0.97 | 88.55 | 85.73 |
| C187 | 77 | 1 | MEDULLA | 0.6 | 81.2 | 0.14 | 0.97 | 7.71 | 7.50 |
| C188 | 77 | 1 | HIPPOCAMPUS | 1.1 | 133.8 | 0.14 | 0.99 | 6.25 | 6.17 |
| C189 | 77 | 1 | BRODMANN 4 | 1.5 | 183.8 | 0.11 | 0.96 | 3.10 | 2.97 |
| C190 | 77 | 1 | HIPPOCAMPUS | 0.6 | 72.5 | 0.14 | 0.70 | 13.85 | 9.75 |
| C191 | 77 | 1 | ANT CINGULATE | 1.0 | 121.3 | 0.16 | 0.95 | 8.99 | 8.52 |
| C192 | 77 | 1 | CEREBELLUM | 1.7 | 207.5 | 0.03 | 0.98 | 2.25 | 2.22 |
| C193 | 77 | 1 | TECTUM | 0.6 | 70.0 | 0.13 | 1.00 | 7.47 | 7.45 |
| C194 | 77 | 1 | OCCIPITAL | 0.7 | 85.0 | 0.13 | 0.97 | 8.27 | 8.02 |
| C195 | 1 | 0 | OLFACT BULB | 0.3 | 35.0 | 0.34 | 0.96 | 27.87 | 26.68 |
| C196 | 1 | 0 | OLFACT BULB | 0.8 | 97.5 | 0.13 | 1.00 | 6.63 | 6.63 |
| C197 | 1 | 1 | OLFACT BULB | 0.5 | 61.2 | 0.10 | 0.95 | 9.45 | 8.99 |
| C198 | 1 | 1 | OLFACT BULB | 0.4 | 48.8 | 0.27 | 0.84 | 21.15 | 17.68 |
| C199 | 1 | 0 | OPTIC CHIASM | 0.1 | 10.0 | 1.48 | 0.97 | 112.69 | 108.86 |
| C200 | 1 | 0 | VAGUS NERVE | 0.3 | 32.5 | 0.62 | 0.96 | 35.54 | 34.13 |
| C201 | 1 | 0 | HYPOPHYSIS | 0.1 | 12.5 | 0.81 | 0.99 | 42.35 | 42.11 |

***Supplemental Table 2*:** Forensic autopsy subjects with APOE and neuropathological diagnosis. Cases were examined with H&E, PHF-tau8 phosphorylated at Ser199-202-Thr205, α-synuclein phosphorylated at Ser-129, LB509 and TDP-43 mab2G10 and rabbit polyclonal Ab recognizing N-terminal TDP-43.ID# corresponds to the magnetic studies identification data in Suppl Table 1.

| ID# | AGE | SEX | APOE | Htau stage | Abeta Phase | Htau NTs | HtauNFT | OBαS | LC Htau | SN Htau | SNαS | TDP-43 BRAINSTEM | |
| --- | --- | --- | --- | --- | --- | --- | --- | --- | --- | --- | --- | --- | --- |
| C001 | 25 | 1 | 0 | 2 | 2 | 2 | 2 | 2 | 0 | 0 | 1 | 0 |  |
| C 002 | 55 | 1 | 0 | 3 | 2 | 1 | 1 | 0 | 0 | 0 | 0 | 0 |  |
| C003 C004 | 55 | 1 | 0 | 5 | 4 | 2 | 2 | 1 | 0 | 0 | 0 | 0 |  |
| C005 | 90 | 0 | 0 | 4 | 3 | 1 | 1 | 1 | 1 | 1 | 1 | 2 |  |
| C006 | 41 | 1 | 0 | 5 | 3 | 1 | 1 | 1 | 0 | 1 | 1 | 1 |  |
| C007 | 29 | 0 | 0 | 2 | 2 | 1 | 1 | 1 | 0 | 1 | 1 | 1 |  |
| C008 | 39 | 1 | 0 | 3 | 2 | 2 | 1 | 1 | 0 | 0 | 0 | 0 |  |
| C009 | 53 | 1 | 0 | 4 | 3 | 1 | 2 | 1 | 1 | 1 | 1 | 1 |  |
| C010 | 27 | 1 | 0 | 2 | 2 | 1 | 1 | 1 | 0 | 2 | 1 | 1 |  |
| CO11 | 65 | 1 | 0 | 4 | 3 | 1 | 1 | 1 | 1 | 1 | 1 | 0 |  |
| CO12-CO19 | 3 | 1 | 0 | 2 | 0 | 1 | 1 | 1 | 0 | 1 | 0 | 0 |  |
| CO20-CO27 | 1 | 1 | 0 | 1 | 2 | 1 | 0 | 0 | 0 | 1 | 0 | 1 |  |
| CO28-CO35 | 24 | 1 | 0 | 2 | 2 | 1 | 1 | 1 | 0 | 0 | 0 | 0 |  |
| CO36-CO40 | 13 | 0 | 0 | 2 | 2 | 1 | 0 | 1 | 0 | 1 | 1 | 0 |  |
| CO41-CO48 | 16 | 1 | 0 | 2 | 2 | 1 | 1 | 1 | 0 | 1 | 0 | 1 |  |
| CO49-C056 | 25 | 0 | 0 | 2 | 2 | 1 | 0 | 1 | 0 | 1 | 0 | 0 |  |
| C085-C087 | 17 | 1 | 0 | 1 | 2 | 0 | 0 | 0 | 0 | 0 | 0 | 0 |  |
| C088-C093 | 45 | 1 | 0 | 4 | 3 | 1 | 1 | 1 | 1 | 1 | 0 | 0 |  |
| C094-C099 | 54 | 1 | 0 | 3 | 2 | 1 | 0 | 0 | 1 | 1 | 0 | 0 |  |
| C0100-104 | 17 | 1 | 0 | 3 | 2 | 1 | 1 | 1 | 1 | 1 | 0 | 1 |  |
| C105-C112 | 35 | 1 | 0 | 2 | 2 | 1 | 1 | 1 | 1 | 1 | 0 | 0 |  |
| C113-C120 | 17 | 1 | 0 | 2 | 2 | 0 | 0 | 0 | 0 | 1 | 0 | 0 |  |
| C121-C126 | 14 | 1 | 0 | 2 | 2 | 1 | 1 | 1 | 1 | 1 | 0 | 0 |  |
| C127-134 | 58 | 1 | 0 | 3 | 2 | 1 | 0 | 0 | 1 | 1 | 0 | 0 |  |
| C135-C138 | 22 | 0 | 0 | 2 | 2 | 1 | 1 | 1 | 1 | 1 | 1 | 1 |  |
| C140-C145 | 31 | 1 | 0 | 2 | 2 | 1 | 1 | 0 | 0 | 0 | 0 | 0 |  |
| C146-C150 | 27 | 1 | 0 | 2 | 2 | 1 | 1 | 1 | 0 | 0 | 0 | 0 |  |
| C151-CC155 | 20 | 1 | 1 | 2 | 2 | 2 | 2 | 1 | 0 | 1 | 1 | 0 |  |
| C156-C160 | 63 | 0 | 0 | 4 | 3 | 2 | 1 | 0 | 0 | 1 | 1 | 0 |  |
| C161-C171 | 36 | 1 | 1 | 5 | 3 | 0 | 0 | 0 | 2 | 3 | 1 | 1 |  |
| C172-C174 | 72 | 1 | 0 | 4 | 3 | 1 | 1 | 1 | 1 | 1 | 1 | 1 |  |
| C175-C179 | 18 | 1 | 0 | 2 | 2 | 1 | 1 | 0 | 1 | 1 | 1 | 0 |  |
| C180-C186 | 21 | 1 | 0 | 3 | 2 | 1 | 1 | 1 | 1 | 0 | 1 | 1 |  |
| C187-C194 | 77 | 0 | 0 | 4 | 3 | 2 | 1 | 2 | 2 | 1 | 1 | 2 |  |

Gender: 0=female, 1=male. APOE 0=3/3, 1=3/4

AD staging pτ Stage: 0=absent, 1= pre-tangle stages a-c, 2= pre-tangle stages 1a,1b, 3=NFT stages I, II, 4=NFT stages III-IV, 5=NFT stages V-VI

AD staging Aβ Phase: 0=absent, 1=basal temporal neocortex, 2=all cerebral cortex, 3=subcortical portions forebrain, 4=mesencephalic components, 5=Reticular formation and cerebellum.

Substantia nigrae pτ was evaluated as none=0, pre-tangles, positive neurites, and tangles using the PHF-tau8 phosphorylated at Ser199-202-Thr205 =1(Innogenetics, Belgium, AT-8 1:1000).

Substantia nigrae α-S was evaluated as none=0 and neuronal immunoreactive (IR) aggregates in the somato-dendritic compartment, cytoplasmic inclusions, core-halo Lewy bodies and dystrophic neurites (Lewy neurites)=1, using α-synuclein phosphorylated at Ser-129, LB509 (In Vitrogen, Carlsbad, CA 1:1000)

* Brainstem TDP-43 using two Ab was evaluated as none=0 and dash-like IR particles in the vicinity of the cell nucleus, with or without complete loss of nuclear TDP-43 expression and somatic skein-inclusions=1, using mab2G10 (Roboscreen GmbH, Leipzig, Germany 1:1000) and Proteintech TDP-43 rabbit polyclonal antibody recognizing the N-terminal TDP-43 (Proteintech #10782-2-AP).
